# Abundance-based detectability in a spatially-explicit metapopulation: a case study on a vulnerable beetle species in hollow trees

**DOI:** 10.1007/s00442-018-4220-5

**Published:** 2018-07-31

**Authors:** Fabien Laroche, Heidi Paltto, Thomas Ranius

**Affiliations:** 1 Irstea, UR EFNO, Domaine des Barres, 45290 Nogent-sur-Vernisson, France; 2 Department of Ecology, Swedish University of Agricultural Sciences, Box 7044, 75007 Uppsala, Sweden

**Keywords:** Occupancy, SPOM, Likelihood, Missing data, Colonization

## Abstract

In many fragmented habitats, the detectability of a population in a habitat patch closely depends on the local abundance of individuals. However, metapopulation studies rarely connect abundance and detectability. We propose a framework for using abundance-based estimates of detectability in the analysis of a spatially-explicit stochastic patch occupancy model (SPOM). We illustrate our approach with the example of *Tenebrio opacus*, a beetle inhabiting hollows in old trees, and have based it on a 6-year monitoring programme of adult beetles in an area harbouring a high density of old oaks. We validated our abundance-based methodology by showing that the estimates of detectability were positively and significantly correlated with those obtained from presence/absence data (Pearson *r* = 0.54, *p* < 2E−16) in our study system. We further showed that the height of the hollow on the tree and the area of its entrance hole, the living status and girth of the host tree, and the time of survey significantly affected the detectability of beetle populations. Median detectability was 51% for one survey. The SPOM analysis revealed a high but heterogeneous extinction risk among trees, suggesting a metapopulation dynamics between the “classic” and “mainland–island” paradigms. However, it also indicated unexplained beetle colonization of trees in our study, despite the fact that we included limited detectability in our estimation procedure. This may have been due to the cryptic larval stage of *T. opacus* and may thus invalidate the use of a classic SPOM in our study system.

**Electronic supplementary material:**

The online version of this article (10.1007/s00442-018-4220-5) contains supplementary material, which is available to authorized users.

## Introduction

Many species rely on habitats that are fragmented, either naturally or due to anthropogenic habitat loss. Their long-term persistence depends on their ability to survive in the habitat patches they occupy and to colonize empty patches. Metapopulation theory offers a framework to study patch occupancy dynamics as a product of colonization and extinction rates (Hanski 1998). The metapopulation concept is valid for species which form subpopulations in habitat patches under certain conditions: the subpopulations must show asynchronous dynamics, must be extinction prone and must be able to reappear after extinction through recolonization (Hanski et al. 1995). Under these assumptions, a species will decline if the number of colonization/recolonization events does not balance the extinctions of existing local populations. Many species do not fit within the metapopulation framework (Fronhofer et al. 2012), but for those that do (e.g. various invertebrate systems; Hanski et al. 1994; Lamy et al. 2013), estimating colonization and extinction rates is a critical input from a conservation perspective. Stochastic patch occupancy models (SPOMs) provide a powerful way to assess these rates using spatiotemporal data on presence/absence in patches (Verboom et al. 1991; Hanski 1994; Eber and Brandl 1996; Moilanen 1999; O’Hara et al. 2002; Ter Braak and Etienne 2003).

The quality of both presence/absence and abundance data is crucial when fitting a SPOM. In particular, false absences due to limited detectability of the target species can lead to biased estimates of colonization and extinction rates and to an underestimation of the strength of the distance limitation on colonization (Moilanen 2002). Repeating occupancy surveys in the same patch makes it possible to estimate the detectability of the populations (Kery 2002; MacKenzie et al. 2002; Ward et al. 2017) and to control for undesirable biases on colonization/extinction estimates (MacKenzie et al. 2003). This technique has been applied both in spatially-implicit (MacKenzie et al. 2003; Lamy et al. 2013) and spatially-explicit SPOMs (e.g. Sutherland et al. 2014; Chandler et al. 2015).

However, integrating classic occupancy-based estimates of detectability into a spatially-explicit metapopulation framework has some limitations. Occupancy data (i.e. presence/absence) have limited statistical power, which renders the joint estimation of detectability and spatially-explicit metapopulation dynamics possible only on very large datasets. In particular, although accounting for the effect of site environmental features on detectability in a spatially-explicit metapopulation is theoretically possible (MacKenzie et al. 2003), it is challenging in practice. For instance, in a 6-year monitoring programme of leopard frog metapopulations across 41 sites in Arizona, Chandler et al. (2015) proposed a hierarchical Bayesian framework to account for temperature and wind effects on detectability. However, their statistical analysis showed that occupancy data did not have enough power to determine whether these two environmental features had effects significantly different from 0 upon detectability.

Here, we propose an alternative way to introduce limited detectability in SPOM analyses. Because the density of a population positively affects its detectability (Delaney and Leung 2010; McCarthy et al. 2013), we suggest estimating detectability with observed abundance data (i.e. an “abundance-based” estimation of detectability), instead of using repeated surveys (i.e. an “occupancy-based” estimation of detectability). Abundance-based estimates of detectability can be obtained separately, then integrated into the SPOM analysis of metapopulation occupancy data.

We illustrate our approach on *Tenebrio opacus* (Duftschmid, 1812), a beetle confined to hollow trees, in the province of Östergötland, southeast Sweden, which is one of the few remaining landscapes in Northern Europe with a high density of old oaks (Antonsson and Wadstein 1991). In oaks, the first hollows are typically formed when they are 200–350 years old (Ranius et al. 2009). From a conservation perspective, old oaks are declining at a global level due to land use changes (Lindenmayer et al. 2012) and *T. opacus* is now classified as “vulnerable” on the Swedish red list (Swedish Species Information Centre 2015). This calls for a better knowledge of the colonization/extinction dynamics of *T. opacus* in hollow tree networks. Studies on another beetle species inhabiting hollow trees (Ranius 2007) suggest that this type of system may borrow from three distinct metapopulation paradigms: classic metapopulations and mainland–island metapopulations (as defined by Fronhofer et al. 2012), and habitat-tracking metapopulations. The difference between these paradigms mainly lies in the pattern of local extinctions, a feature that has never been studied on *T. opacus*. We thus had no a priori assumption about this point in our study. In Östergötland, *T. opacus* occurrence in hollow trees is known to be positively related to the number of occupied trees in a radius of about 800 m (Ranius et al. 2010). This suggests that the colonization rate decreases with the distance from dispersal sources to such an extent that suitable but isolated trees are more likely to become unoccupied. This has also been observed in many other beetle species inhabiting hollow trees (Ranius et al. 2010; Bergman et al. 2012; Kadej et al. 2016), with relatively small spatial scale of effect (median among species in Ranius et al. (2010) is 400 or 900 m, depending on habitat metrics used; Bergman et al. (2012) report 500 m). The spatial scale of effect is generally assumed to be related to the typical dispersal distances of the species (Jackson and Fahrig 2012). However, there has been no direct assessment of distance limitation in colonization based on a temporal survey for *T. opacus*.

The aim of our study was threefold. We first wished to validate an approach connecting density to detectability. To do so, we compared abundance-based estimates of detectability to occupancy-based estimates independently computed from repeated surveys of presence/absence. We expected a good match and no bias between the two quantities. Second, we explored whether abundance data had enough power to detect significant environmental effects upon detectability. We expected these significant environmental effects to correspond to those identified as drivers of *T. opacus* presence/absence in previous studies (Ranius et al. 2011). Third, we tested whether introducing our abundance-based estimates of detectability into a SPOM analysis of *T. opacus* occupancies improved metapopulation estimates compared to an analysis assuming perfect detection. The SPOM we used was a spatially-realistic metapopulation model that simultaneously allowed for distance-limited colonization among trees and “spatially-unstructured colonization”. Spatially-unstructured colonization reflects the fact that individuals coming from outside the metapopulation system can colonize a vacant tree; the colonization rate is assumed to be equal for all trees. If *T. opacus* is hard to detect, assuming perfect detection would generate many false absences and show both significant unstructured colonization and little distance limitation for colonization. However, accounting for limited detectability should rectify this: spatially-unstructured colonization should be non-significant in this case, and distance limitation for colonization should be stronger. In addition, any residual effect of unstructured colonization when accounting for limited detectability (i.e. feeding the SPOM with abundance-based detectability estimates) is then evidence of unknown sources of colonists, i.e. the studied metapopulation is not closed.

## Methods

**Fig. 1:**
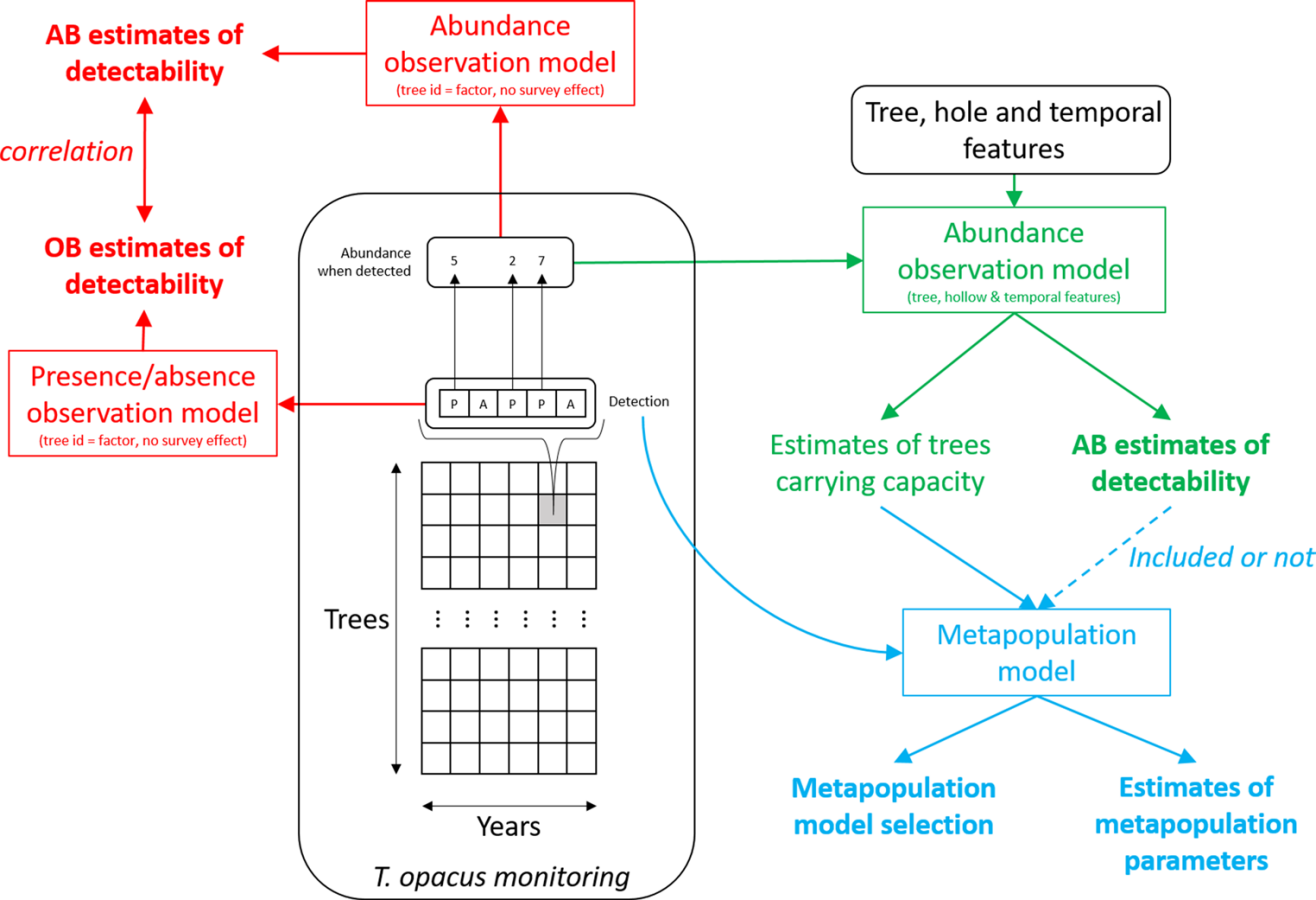
Overview of our framework for combining abundance-based estimates of detectability and metapopulation analysis of *T. opacus*. Frames with sharp angles indicate modelling parts, while frames with round corners contain data. All the analyses are based on the *T. opacus* monitoring dataset, which consists of an array of tree × year observations. Each tree × year observation (e.g. the grey cell in the grid) consists of a series of surveys. In the figure, the grey cell represents five surveys, presented in a row inside a curly bracket. Each survey leads either to detection (P for presence in the row vector) or no detection (A for absence in the row vector). For surveys where the presence was detected, we also show the number of individuals observed (presented above the detection outcome in the figure). We distinguished three main steps in the analysis: comparing detectability estimates obtained from either presence/absence or abundance-when-present data (in red); computing detectability estimates using abundance-when-present data (in green); estimating metapopulation dynamics with and without accounting for detectability (in blue)

Our framework consisted of four steps: (1) *T. opacus* monitoring in the field; (2) comparing abundance-based estimates of detectability with more classic occupancy-based ones; (3) deriving abundance-based estimates of detectability for all field surveys and carrying capacity estimates for all the studied trees; and (4) deriving metapopulation dynamics estimates and testing the effect of including detectability (Fig. 1).

### *Tenebrio opacus* monitoring

We monitored an area of 18.3 km^2^ in the surroundings of Bjärka Säby, in the province of Östergötland in south-eastern Sweden. This corresponds to about ten times the area delineated by a circle with an 800-m radius, which has previously been identified as the spatial scale of response for occurrence of *T. opacus* (Ranius et al. 2010). Consequently, we expected to find colonization distance limitations at our study site. The density of hollows on old oaks was particularly high at the centre of our area of study, where there were known occurrences of *T. opacus* (Ranius 2002). We carefully searched through the entire site to find potential host oaks in the field. We recorded and monitored all the standing oaks harbouring an entrance hole with a diameter > 10 cm. A total of 338 hollow trees were included in the analysis.

Both the adults and larvae of *T. opacus* dwell in wood mould, i.e. loose material in tree hollows made up of rotten wood. We were able to count the adults relatively easily since they are visible on the tree trunk at night time, typically close to the entrance of a hollow. We used a torch and a ladder (about 5 m high) to carefully inspect trees during surveys. Each summer (from beginning June to end September), there were between zero and ten surveys per tree from 2007 to 2012. The number of surveyed trees (i.e. at least one inspection) varied from year to year with the most sustained monitoring efforts in 2009 and 2010 (Table 1).

**Table 1:** Survey results for the *T. opacus* metapopulation in the Bjärka Säby area

Year	Number of surveyed trees	Number of surveys	Number of collected individuals	Number of marked individuals	Number of recaptured individuals
2007	47	89	73	51	7
2008	104	435	157	107	22
2009	294	1989	553	403	103
2010	329	1475	327	246	45
2011	21	63	45	0	0
2012	34	100	107	0	0

We captured, marked, counted and released the adult individuals at each survey. Even though there are some differences in morphology between males and females, we were not able to identify the sex for all the individuals counted. A fraction of the captured beetles was marked from 2007 to 2010, and a basic analysis of the capture–mark–recapture data revealed two important pieces of information for this study. First, we recaptured some adults for up to three seasons (Online Resource 1: Fig. S1.1), showing that *T. opacus* can survive in its adult form for at least 2 years. Second, > 70% of the recaptured beetles were recaptured on the same tree (Online Resource 1: Table S1.1), whether recapture occurred later the same year or a different year; this tends to suggest that beetles on different trees constitute truly distinct populations and that a panmictic system does not exist in the study area.

### Comparing abundance-based and occupancy-based detectability estimates

In all our analyses, we assumed that beetle populations on individual trees could appear and disappear from one year to the next, but that occupancies and abundance were constant for a given year. When a population is present in tree *i* at year *t*, we assumed that the number of beetles *N*_its_ observed at the *s*th survey followed a geometric distribution (see Online Resource 2 for justification). For this step of the analysis only, we further assumed that the geometric distribution depended on tree *i* and year *t* but not on survey *s*. This gives:

$$ {\mathbb{P}}\left( {N_{its} = n} \right) = \left( {1 - g_{it} } \right)^{n} g_{it} , $$

where *g*_it_ is a positive parameter specific to the considered tree × year combination. The detectability in any survey for year *t* in tree *i* thus verifies *φ*_it_ = 1 − *g*_it_.

Using abundance data for surveys where *N*_its_ > 0, we performed a Bayesian estimation of detectability *φ*_it_ (see Online Resource 3). For each abundance-based detectability estimate *φ*_it_ obtained, we computed an alternative detectability estimate based on the presence/absence data in the same tree × year combination (which we called “occupancy-based”; see Online Resource 3). Thus, we could check whether these two independent ways of estimating detectability showed a good degree of correlation.

### Deriving abundance-based estimates of detectability for all surveys and estimating carrying capacity for all trees

We measured tree features that earlier studies had shown affect the occurrence of beetle species confined to hollow oaks (Ranius 2002). For every tree, we recorded (1) girth, (2) living status (alive or dead) and (3) sun exposure. For every entrance hole, we estimated (4) the height on the trunk and (5) a proxy of the area of the entrance hole (computed from the height and width of the hole). For trees with several cavities (some trees harboured up to eight holes), we used the average height and the total area of the entrance holes. Because there were strong correlations between hollow features and tree features (for instance, larger trees had larger holes), we did not directly include tree features as explanatory variables in our analysis. Instead, we first built generalized linear models of tree features using hollow features as covariates (Online Resource 2) and then included the residual variation of tree features as an explanatory variable in our abundance observation model (Fig. 1).

We explored how tree features, hollow features and sampling context affect population detectability and tree-carrying capacity. We continued to assume that when a population was present in tree *i* at year *t*, the number of beetles *N*_its_ observed at the sth survey of year *t* in tree *i* followed a geometric distribution (see Online Resource 2). However, for this step of the analysis and for the rest of the study, we assumed that the distribution depended not only on tree *i* and year *t,* but also on survey *s*. This gives:

$$ {\mathbb{P}}\left( {N_{\text{its}} = n} \right) = \left( {1 - g_{\text{its}} } \right)^{n} g_{\text{its}} , $$

where *g*_its_ is a positive parameter specific to the considered tree × year × survey combination. The detectability at survey *s* of year *t* in tree *i* thus verifies *φ*_its_ = 1 − *g*_its_. We then built an environmental model of *g*_its_ as follows:

1 $$ {\text{logit}}\left( {g_{\text{its}} } \right) = \mu + \theta_{i} + \tau_{\text{its}} , $$

where *θ*_*i*_ is a linear combination of the features of tree *i* (including hollow features) and *τ*_its_ is a linear (or quadratic for dates) combination of date, time and temperature at the *s*th visit of year *t* at tree *i*. We only used surveys where *N*_its_ > 0 for calibration and we performed a downward model selection based on log-likelihood ratio tests.

We then extrapolated the final model to surveys where *N*_its_ = 0, and obtained a predicted value of *g*_its_ for all the surveys in our dataset. We computed the detectability in tree *i* during year *t* (i.e. the probability of detecting an existing population in tree *i* in at least one survey of year *t*), called Φ_it_, as follows:

2 $$ {\varPhi }_{\text{it}} = 1 - \mathop \prod \limits_{s = 1}^{{s_{\text{it}} }} g_{\text{its}} $$

where *s*_it_ is the number of surveys in tree *i* during year *t* (when *s*_it_ = 0, ${\varPhi }_{it} = 0$). Φ_it_ is the detectability we used to compute the pseudo-likelihood of observed occupancy data in the metapopulation model described in the next section.

We modelled the carrying capacity of each tree (*K*_1_, …, *K*_*N*_) as follows:

3 $$ K_{i} = - { \log }\left( {\frac{{{\text{e}}^{{\mu + \theta_{i} }} }}{{1 + {\text{e}}^{{\mu + \theta_{i} }} }}} \right), $$

where *μ* and *θ*_*i*_ originate from extrapolating the environmental model (1) to all the trees in the study area.

### Deriving metapopulation estimates and testing the effect of including detectability

We developed a spatially-explicit SPOM to estimate metapopulation parameters from our *T. opacus* occupancy dataset (Fig. 1) with an approach similar to Chandler et al. (2015). We described the colonization–extinction dynamics of *T. opacus* at discrete time steps corresponding to surveyed years. *z*_*i*,*t*_ denotes the occupancy of tree *i* at year *t* (*z*_*i*,*t*_ = 1 if tree *i* is occupied by a population at time *t*; 0 otherwise). We used the following metapopulation model with a pseudo-rescue effect (Hanski 1999, p. 60; Chandler et al. 2015):

4 $$ z_{i,t} \sim \gamma_{i,t - 1} \left( {1 - z_{i,t - 1} } \right) + \left( {1 - \left( {1 - \gamma_{i,t - 1} } \right)\varepsilon_{i,t - 1} } \right)z_{i,t - 1} , $$

where *ɛ*_*i*,*t*−1_ is the probability that a population occupying tree *i* at time *t* − 1 went extinct between *t* − 1;  and *t*, *γ*_*i*,*t*−1_ is the probability that if tree *i* was empty at time *t* − 1,  it was colonized between *t* − 1 and *t*.

We assumed that the extinction rate in tree *i* was related to the tree-carrying capacity *K*_*i*_ through $\varepsilon_{i,t} = {\text{e}}^{{ - sK_{i} }}$ (Ovaskainen 2001), where *s* is a parameter measuring the general survival abilities of *T. opacus* populations. Colonization probability was modelled as follows:

5 $$ \gamma_{i,t - 1} = 1 - {\text{e}}^{{ - c_{\text{out}} }} \mathop \prod \limits_{{\begin{subarray}{*{20}c} {j = 1} \\ {j \ne i} \\ \end{subarray} }}^{N} \left( {1 - \rho_{i,j,t} } \right), $$

where $\rho_{i,j,t}$ is the probability that an individual from tree *j* at time *t* − 1 colonized the empty tree *i* between *t* − 1 and *t*. More precisely,

6 $$ \rho_{i,j,t} = \left[ {1 - { \exp }\left( { - cK_{j} e^{{ - \alpha d_{ij} }} } \right)} \right]z_{j,t - 1} , $$

where *c* is a parameter measuring the per-capita intensity of *T. opacus* propagule production, *α* measures the increase of propagule mortality with dispersal distance (i.e. the “strength” of the distance limitation in colonization), and *d*_*ij*_ is the Euclidean distance from tree *i* to tree *j*. *c*_out_ quantifies “spatially-unstructured colonization”, which is the intensity of the propagule flux coming from unknown sources distinct from surrounding oaks in the study area. *c*_out_ drives the probability (equal to $$ 1 - {\text{e}}^{{ - c_{\text{out}} }} $$) that if a given tree *i* is empty at time $$ t - 1, $$ it is colonized from these unknown sources between *t* − 1 and *t*. The vector of the parameters describing metapopulation dynamics is noted as $$ {\varTheta } = (c, s,\alpha ,c_{\text{out}} ) $$.

The shape of distance limitation used in Eq. (6) is exponentially decreasing, which corresponds to a thin-tail colonization kernel (i.e. little chance of extreme events in terms of colonization distance within the studied area). We repeated the analyses presented below with a fat-tail colonization kernel, to check whether our conclusion was robust to the presence of extreme events in terms of colonization distance within the studied area (see Online Resource 5).

We estimated Θ by maximizing a pseudo-likelihood function from observed tree occupancy data (see Online Resource 4 for details). The core assumption we made was to consider that *real* occupancies of trees at year *t* were independent one from another given *observed* occupancies in the past. Our pseudo-likelihood function uses detectability over a whole year (Φ_*it*_ parameters) as an input. We performed a first estimation assuming perfect detection: during maximization, we fed the pseudo-likelihood function with Φ_*it*_ parameters equal to 1 if there was at least one survey in the tree × year combination; we used Φ_*it*_ parameters equal to 0 otherwise. We then repeated the estimation procedure with limited detectability included: during maximization, we fed the pseudo-likelihood with Φ_*it*_ estimates obtained in the previous section. We further assessed whether the sampling effort while monitoring *T. opacus* occupancies modulated the effect of introducing detectability in our case study: we performed the same estimation procedure of metapopulation parameters with and without accounting for detectability, but using degraded datasets where we only kept one survey per tree × year combination.

We used simulated datasets to obtain the bias, variance/covariance matrix and mean square error associated to our estimation procedure (see Table 3 and Online Resource 4). We tested whether the estimates of $c, s,\alpha ,c_{\text{out}}$ were significantly different from 0 by comparing the full model (obtained from the pseudo-likelihood above) with four sub-models where some parameters were constrained to 0 and the others were estimated: (1) a closed Levins model (“ClosedLM”; corresponding to *α* = 0 and *c*_out_ = 0); (2) a closed spatially-realistic Levins model (“ClosedSRLM”; corresponding to *c*_out_ = 0); (3) a propagule rain model (“PropRain”; corresponding to *c* = 0 and *α* = 0); and (4) an open Levins model (“OpenLM”; corresponding to *α* = 0). We compared these models using a pseudo-AIC obtained from the pseudo-likelihood for model selection.

## Results

On average, we observed 0.3 individuals per survey event per tree (Online Resource 2). The maximum number of observed individuals in a single survey for a single tree was 21. In 145 trees of the area of study, we detected *T. opacus* individuals at least once over the whole monitoring programme. For 139 of these trees, surveys were performed at two distinct years or more, which allowed searching for apparent colonization and extinction events. We identified 34 trees where a population was detected at all surveyed years (i.e. no extinction), 60 trees where at least one apparent colonization occurred, 69 trees where at least one apparent extinction occurred, and 8 trees where at least one apparent extinction–recolonization cycle occurred.

### Comparing abundance-based and occupancy-based detectability estimates

**Fig. 2:**
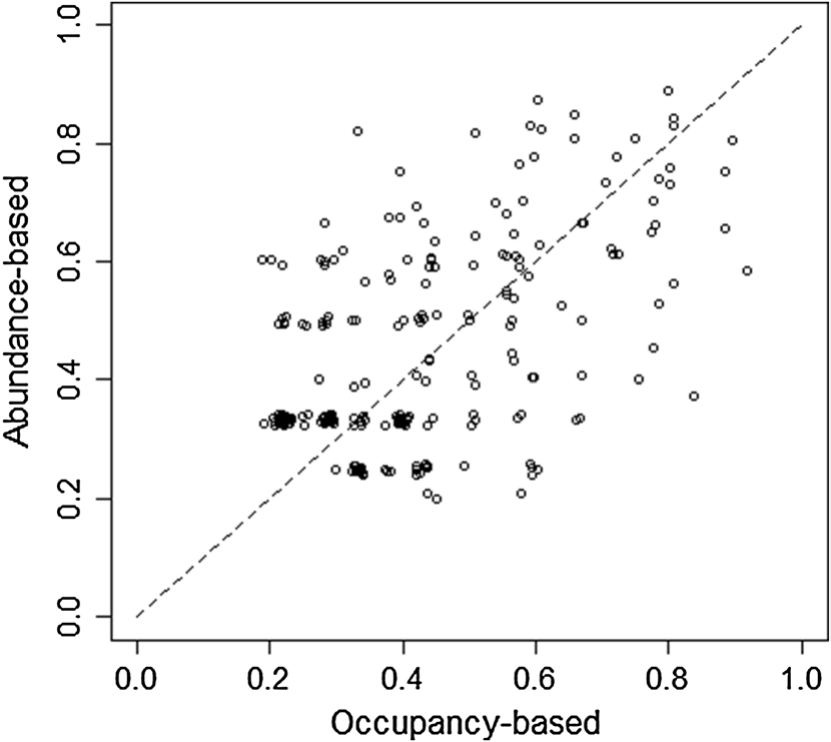
Comparing occupancy-based and abundance-based estimates of detectability in a single survey. Only tree × year combinations where at least one *T. opacus* individual was observed were included in this analysis. Since many points were exactly superimposed, we added a small random perturbation between + 0.01 and − 0.01 on the two coordinates of each points. See Online Resource 3 for the computation of estimates, based on a Bayesian approach. We added the first diagonal *y* = *x* on the diagram as a black dashed line

We detected *T. opacus* individuals in 251 tree × year combinations (i.e. detection in at least one survey of the given year in the given tree). We could, therefore, compute 251 pairs of detectability estimates in a single survey (either occupancy- or abundance-based; Fig. 2). The Pearson correlation between the two estimates equalled 0.54 (greater than 0 with *p* < 2E−16). This value is among the highest possible given the variance of the detectability estimates (see Online Resource 3). Occupancy-based estimates were slightly but significantly greater than abundance-based estimates on average (difference equalled 0.02; an asymptotic *t* test yielded *p* = 0.04). The standard deviation of the difference between the two estimates was 0.16.

### Environmental drivers of population detectability

Trees with larger entrance holes located closer to the ground yielded significantly higher numbers of observed individuals. After controlling for hollow features (Online Resource 2), living trees with a larger girth yielded higher numbers of observed individuals (Table 2). Sun exposure, on the other hand, had no detectable effect on the number of observed individuals. Date of survey per se did not significantly affect the numbers of observed individuals but after controlling for date, we found that surveys occurring later in the night yielded a larger number of observed individuals. We found no effect of temperature on the survey after controlling for date and time. In all, these variables explained a limited proportion of the variation in the numbers of observed individuals: the selected model for number of observed individuals had a McFadden *R*^2^ of 0.12.

**Table 2:** Effects of tree and hollow features on the number of observed individuals during successful detection events

Explanatory variable	Standardized effect	Std. error	*p* value
Entrance hole area	0.29	0.067	2e−05
Mean entrance hole height	− 0.27	0.070	1e−04
Tree living status (residual)	0.29	0.070	4e−05
Tree girth (residual)	0.20	0.065	2e−03
Time of survey	0.20	0.062	7e−04

We report the coefficient of the standardized tree features. Effects are multiplied by − 1 to ensure that a positive coefficient means a positive effect on count data. 145 trees were included in the analysis with a total of 515 visits analyzed. See the main text and Online Resource 2 for details on the model and the covariates

**Fig. 3:**
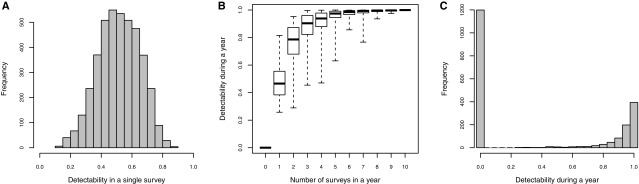
*T. opacus* detectability in a single survey for a whole year. **a** Histogram of detectability with a single survey, for all trees and all years. **b** Detectability for a whole year depending on the number of surveys. Boxes present the distribution of detectability for various numbers of surveys in the year. Boxes are computed with predicted values from the model presented in Table 2. Whiskers encompass 100% of the values and box boundaries present first and third quartiles. **c** The distribution of year-scale detectability among all the possible tree × year combinations in our study

All the effects evidenced above also apply to detectability for a single survey. Variations in tree features and survey time yielded wide variations in abundance-based estimates of detectability among survey events (Fig. 3a). The median detectability in a single survey was 0.51, meaning that for half of the surveys performed, a population, when present, had less than a 51% chance of being observed. Detectability in tree *i* during year *t* (Φ_it_) depended on the corresponding number of surveys. Six surveys or more per tree per year yielded a detectability for the whole year above 80% for all the concerned trees (Fig. 3b). Three surveys or less per tree per year yielded a detectability for the whole year below 80% for more than 25% of the concerned trees (Fig. 3b). In all, of the 2028 tree × year combinations possible during our monitoring, 59% were actually unsurveyed, 15% had a detectability for the whole year below 0.95, and 26% had a detectability for the whole year above 0.95 for the whole year (Fig. 3c).

### Comparing metapopulation estimates with and without limited detectability

When assuming perfect detection and estimating metapopulation parameters on the full occupancy dataset, we found that all parameters in Θ were significantly different from 0 (i.e. the full model had a lower AIC than any sub-model; Table 3). Including limited detectability did not affect model selection: the full model with all the parameters different from 0 was still selected (Table 3). However, the pseudo-AIC values of the full model and sub-models were systematically lower with limited detectability than when assuming perfect detection, indicated a better fit to data with detectability included.

**Table 3:** Metapopulation model selection based on pseudo-AIC assuming perfect detection and including limited detectability

Model	Estimated parameters	Assuming perfect detection		Including limited detectability	
		Pseudo-likelihood	Pseudo-AIC	Pseudo-likelihood	Pseudo-AIC
Closed LM	*c*, *s*	− 471.1	946	− 462.2	928
Closed SRLM	*c*, *s*, *α*	− 470.5	947	− 461.5	929
Prop. rain	*s*, *c*_out_	− 467.8	940	− 460.3	925
Open LM	*c*, *s*, *c*_out_	− 467.8	942	− 460.3	927
Full model	*c*, *s*, *α*, *c*_out_	− 460.7	**929**	− 453.1	**914**

See “Methods” for sub-model descriptions. In bold, the pseudo-AIC of the selected model

**Fig. 4:**
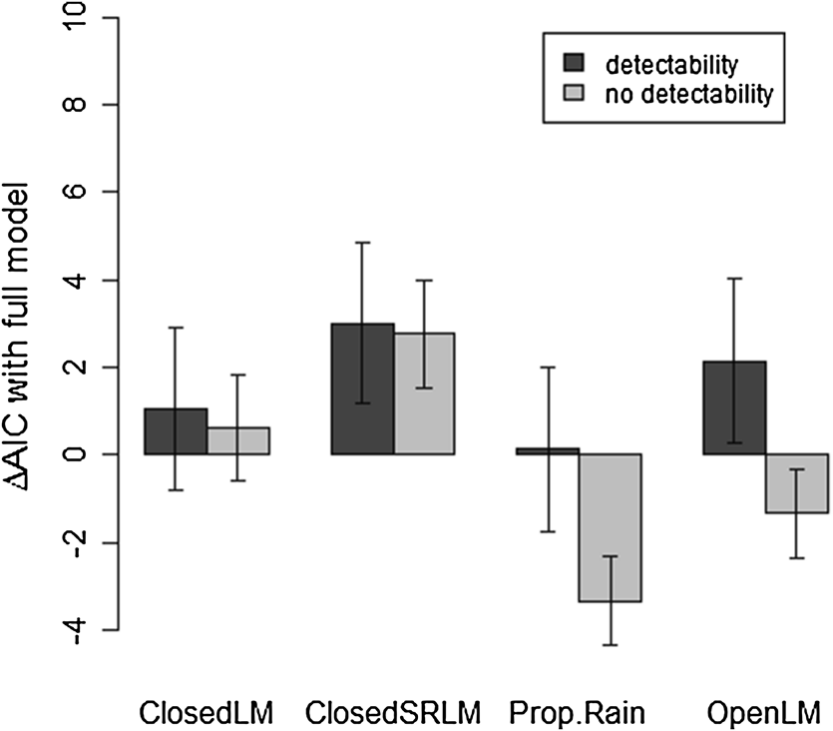
Effect of introducing detectability on the difference in AIC between metapopulation sub-models and the full model when the occupancy dataset was degraded to one survey or less per year. Differences were computed as the AIC of each sub-model minus the AIC of the full model (see main text for definitions of sub-models). These differences were computed on 30 degraded datasets obtained by selecting a single survey at random from each tree × year configuration where surveys were performed. Bars present the average AIC difference across the 30 replicates, and error bars present the standard error of the mean

Considering degraded occupancy datasets yielded different results (Fig. 4). When assuming perfect detection, the propagule rain model (*c*, *α* = 0; unstructured colonization only) had a significantly lower AIC (by three points) than the full model and performed better than any other sub-model (although not significantly better than the open Levins model). By contrast, when limited detectability was included, the full model was identified as the best model (i.e. had the lowest AIC on average) on the degraded datasets, although the difference in AIC with the propagule rain (*c*, *α* = 0; unstructured colonization only) and the closed Levins (*c*_out_, *α* = 0) models was not statistically significant.

Regarding the parameter estimation obtained using the full occupancy dataset, the full metapopulation model with limited detectability yielded estimates not significantly different from those obtained when assuming perfect detection (Table 4). Colonization probability from one year to another (corresponding to *γ*_i_^*^ in Online Resource 4) varied between 0.14 and 0.37 among trees (mean 0.19). According to our *c* and *α* estimates, a tree which was more than 142 m from its nearest neighbour [95% CI of (46 m, 331 m)] had a negligible (< 0.001) probability of being colonized by individuals from another inhabited tree in the area within the year. *c* and *α* estimates showed strong positive correlation in our analysis suggesting that a simultaneous increase of propagule production and distance limitation generates compensating effects and thus does not affect the colonization kernel at the scale of our study area.

**Table 4:** Parameter estimates of the full model assuming perfect detection and including limited detectability

Parameter	Assuming perfect detection		Including limited detectability					
	Estimate	SD	Estimate	SD	Correlation matrix			
					log(*c*)	log(*s*)	log(*α*)	log(*c*_out_)
log(*c*)	− 1.0	0.37	− 0.99	0.40	1	− 0.16	0.95	0.30
log(*s*)	− 0.10	0.062	− 0.074	0.063	–	1	− 0.10	− 0.42
log(*α*)	1.4	0.30	1.4	0.29	–	–	1	0.46
log(*c*_out_)	− 0.86	0.075	− 0.83	0.087	–	–	–	1

Parameters are log-transformed (logarithm to base 10) to limit skewness of their distribution. Standard deviations (and correlation matrix of estimates for the model including detectability) were obtained through simulations of virtual datasets (Online Resource 4)

For the 35 most isolated trees, > 99% of the colonization events were due to unknown colonist sources (i.e. unstructured colonization; representing a yearly colonization probability of 0.14). More generally, the relative importance of unstructured colonization in the total colonization rate varied among trees between 38 and 100%. The yearly probability of extinction varied among trees between 0.23 and 0.86 (average 0.54). For an average hollow tree, this implies that adults maintain 1.8 year on average and have < 5% probability to be present more than 4 years in a row. For trees with the lowest extinction probability, adults maintain 4.2 years on average and have < 5% probability to be present more than 12 years in a row. This latter value extends beyond the number of years covered by our dataset, suggesting that the real persistence of populations in high-quality trees might not be accurately estimated with a 6-year study and could potentially be higher than the estimated value reported here.

## Discussion

The framework developed here proposes a simple procedure to include limited detectability in the analysis of spatially-explicit metapopulation models: first using abundance data in sites where a local population is detected to estimate detectability, second using presence/absence data to estimate colonization and extinction rates only (while accounting for detectability). We emphasize that the two steps rely on independent aspects of the data (abundance when detected vs. presence/absence; Fig. 1), which renders the approach rigorous from a statistical perspective. In addition, site features naturally affect detectability through their effects on the number of observed individuals.

### Comparing abundance-based and occupancy-based detectability estimates

In our system, the correlation between abundance-based and occupancy-based estimates of detectability is positive and strongly significant, which suggests that both approaches capture the same phenomenon. Although abundance-based detectability estimates tend to provide lower values than the occupancy-based estimates, this bias is small (0.02 for probabilities above 0.2). Overall, our analysis provides evidence for the validity of using abundances to estimate detectability.

### Assessing tree, hollow and sampling context effects upon detectability

Thanks to our abundance-based approach, we had enough power to identify tree, hollow and temporal features that significantly affected detectability in our study system. For example, we retrieved tree features shown to affect *T. opacus* presence/absence in previous studies. Several studies (Ranius and Jansson 2000; Ranius 2002; Ranius et al. 2010, 2011) have shown that larger tree girth positively affects *T. opacus* presence in hollows; in our study, tree girth positively affected the detectability of *T. opacus* individuals both directly (through the residual effect of girth) and indirectly (mediated by total hollow area). Similarly, Ranius et al. (2010) documented a higher frequency of *T. opacus* in trees with hollows close to the ground; in our study, the height of the entrance hole negatively affected detectability. The rather low McFadden *R*^2^ value (0.12) of our abundance observation model suggests that *T. opacus* count data have an intrinsically high level of stochasticity or that some additional explanatory variable that we could not measure probably contributed to detectability in our study (e.g. wood mould volume within the hollows). In our framework, we assumed that beetle abundance also conveys information about patch-carrying capacity, and that there is a close link between patch-carrying capacity and population detectability. With these assumptions, our results suggest that the tree girth and the height of the entrance hole may affect carrying capacity, which in turn would affect both the detectability and extinction rate of occurring populations, thus explaining previously proven presence/absence patterns.

Note that carrying capacity and detectability may not be correlated in all metapopulation systems. We believe this to hold for any metapopulation-inhabiting patches of rather limited size that can be extensively covered by surveys; in this case, a higher carrying capacity systematically converts into a higher density of individuals and better detectability. This is reasonable for tree hollow-dwelling beetles, but it may not be the case for butterflies in meadows, frogs in ponds, etc. In these other contexts, a large carrying capacity would not necessarily equate to higher density and better detectability.

### Comparing metapopulation estimates with and without limited detectability

A single survey in a single tree yielded a median detectability of 51% for *T. opacus*, with wide variations among trees. For other species, however, detectability in a single survey has surpassed 95% for epiphytic lichens (Johansson et al. 2010) and 60% for butterflies (Cozzi et al. 2008; Johansson et al. 2017). Therefore, while assuming perfect detection might not bias results for the latter species, one could expect it to generate bias in our study system. Surprisingly, however, this was not the case: the parameter estimation remained unchanged whether we included limited detectability of *T. opacus* or not. This stems from the fact that, given our high sampling effort (up to 10 surveys per tree × year configuration), only 15% of tree × year observations suffered from limited detectability [the rest being either absent (unsurveyed trees) or repeatedly observed]. This proportion was too limited to have affected the outcome of our analysis. Indeed, when artificially degrading our occupancy dataset by keeping only one survey per tree × year combination, we retrieved the expected biased effects of limited detectability upon estimates of metapopulation parameters: assuming perfect detection led to significantly better performance for the models that did not include distance limitation of colonization and included a significant spatially-unstructured colonization rate. Introducing limited detectability contributed to fixing this problem: the full model became the best-fitted model (although not significantly better than some spatially-unstructured sub-models), which was consistent with our results on the unaltered occupancy dataset.

Our results, therefore, suggest that in studies with a high sampling effort like ours, ignoring detectability may have little impact on the estimates of metapopulation parameters. However, even if parameter estimation is little affected, we still found that accounting for detectability improved the fit of the metapopulation model to occupancy data (decreasing pseudo-AIC); this pleads in favour of taking into account limited detectability whatever the sampling effort. In metapopulation studies with a more limited sampling effort than ours (which is frequently the case) or which consider species with lower detectability than *T. opacus* (like many other dead wood-dependent insects that do not leave their hollows at night), taking detectability estimates into account is more important. Our framework provides a simple way of doing so.

### Residual unstructured colonization when accounting for detectability

Our metapopulation estimates of local colonization and extinction rates may suggest that *T. opacus* follow an intermediate dynamics between “classic” and “mainland–island” paradigms of Fronhofer et al. (2012). Indeed, the extinction risk is heterogeneous among trees. Some high-quality trees harbouring large populations have the potential to last longer than the time window of our study while low-quality trees undergo frequent extinction–recolonization dynamics. This echoes the “mainland–island” paradigm. However, the estimated population persistence in high-quality trees is still much shorter than the persistence of typical tree hollows (Ranius et al. 2009), which is in favour of the “classic” metapopulation paradigm. A longer monitoring programme would now be needed to refine the estimation of population persistence in high-quality trees.

Finding significant unstructured colonization, even when accounting for limited detectability, indicates the existence of additional unknown sources of colonization in the system. This could seem similar to the “background deposition” of propagules due to long-distance dispersal events in epiphytic lichen and fungus metapopulations coming from the outside of the studied area (Johansson et al. 2012; Norros et al. 2012). Because the region surrounding our study area has a much lower density of hollow oaks than the study area itself, background deposition would imply frequent long-distance dispersal events from *T. opacus*. However, *T. opacus* occupancy per tree is known to be very low in areas with low densities of hollow oaks within the same region of Sweden (Ranius 2002). Consequently, an extensive background deposition of this species is unlikely and we do not think this effect alone can explain the strength of unstructured colonization in our study system.

Another explanation could be that the arbitrarily-chosen exponential shape for the colonization kernel (Eq. 6) gives a poor fit to occupancy data. The exponential distribution precludes long-distance colonization within the study area. If long-distance colonization events occur, they may appear in our model as unstructured colonization. However, using a different shape of colonization kernel allowing for long-distance colonization events among trees did not reduce unstructured colonization estimate but, on the contrary, increased it (Online Resource 5). In addition, it leads to losing the distance-limited colonization signal, and the global fit to data was worse. We consequently do not retain the lack of extreme colonization distance in the shape of our colonization kernel as a valid explanation for strong unstructured colonization.

A third explanation may be small hollows (with diameters ≤ 10 cm), which we did not include in our survey. However, increasing entrance hole area positively affected the observed number of individuals in our abundance observation model. Thus, small hollows probably played little role in the metapopulation dynamics. Consequently, we have discarded this explanation and proposed instead that unstructured colonization is mainly due to the cryptic larval stage of *T. opacus*. Larval development spans several years and we only observed *T. opacus* adults. Unfortunately, we could not control for larval presence because they live buried in the wood mould inside hollows, and their direct sampling is, therefore, very difficult. As a result, some of the colonization events we observed may have been a consequence of undetected larvae maturing into adults. It is also likely that some extinction events concerned only adults, while a larval population remained present. Indeed, in a hollow tree of average quality, the extinction rates from our model imply that *T. opacus* populations have little chance of lasting more than 4 years, which is an unexpectedly high turnover, hardly higher than the lifespan of a single individual. Not accounting for the cryptic larval stage may thus have led us to overestimate extinction and colonization rates and underestimate spatial limitation of colonization (cf. Moilanen 2002 on detectability). Our conclusion about *T. opacus* metapopulation dynamics being in between the “classic” and “mainland–island” paradigms may thus be fragile. Therefore, we call for a new generation of spatially-explicit SPOMs that account for species with cryptic stages in their life cycle. A way of controlling for this feature would be to incorporate cryptic populations as latent variables in a SPOM, as did Fréville et al. (2013) in a spatially-implicit metapopulation model for a plant species with seed dormancy or Lamy et al. (2013) for a freshwater snail species with estivation behaviour. However, we know of very few empirical applications of this idea in a spatially-explicit context (but see Manna et al. 2017 for a simulation study).

## Conclusion

Because abundance-based detectability estimates can be calculated separately from metapopulation parameters with independent data, they (1) are simpler to handle than occupancy-based detectability estimates, and (2) have the statistical power to unravel the effects of patch environmental conditions upon detectability. In our case study on the tree hollow beetle *T. opacus*, we showed that introducing our detectability estimates into the analysis of occupancy data had little effect on the estimated parameters of metapopulation dynamics when occupancies had been assessed through many repeated surveys, but that the approach did have the potential to improve estimations when the sampling effort was more limited. Therefore, our approach can contribute to improving metapopulation studies with limited budgets for field surveys. We also emphasize the importance of building improved SPOMs robust enough for species with cryptic stages in their life cycle. This is a crucial step toward understanding the population dynamics of a wide array of species.

## Electronic supplementary material

Below is the link to the electronic supplementary material.

Supplementary material 1 (PDF 660 kb)

Supplementary material 2 (PDF 269 kb)

Supplementary material 3 (PDF 106 kb)

Supplementary material 4 (PDF 191 kb)

Supplementary material 5 (PDF 102 kb)

Supplementary material 6 (XLSX 259 kb)
