# Supplementary material for: Abundance-based detectability in a spatially-explicit metapopulation: a case study on a vulnerable beetle species in hollow trees
Source: Oecologia. 2018 Jul 31;188(3):671–82. doi: 10.1007/s00442-018-4220-5 (PMC6208700; doi:10.1007/s00442-018-4220-5)
Supplement: Supplementary file 1 — Supplementary material 1 (PDF 660 kb) [file 442_2018_4220_MOESM1_ESM.pdf]

Online Resource 1: T. opacus capture-mark-recapture results

F. Laroche, H. Pallto and T. Ranius

Table S1.1. Numbers of recapture events in the capture-mark-recapture dataset. Recaptures occurring the same season as the previous detection or the season after, and recaptures occurring in the same tree as previous detection or in a different tree are distinguished. In total 864 individuals were marked. Only 158 individuals were recaptured at least one time. Among them, some could be recaptured up to 6 times, which explains that the number of recapture events (218) is higher than the number of recaptured individuals.

	**Same season**	**Next season**
Same tree	157	35
Other tree	16	10

Figure S1.1. Numbers of consecutive seasons where the 864 marked individuals have been observed.

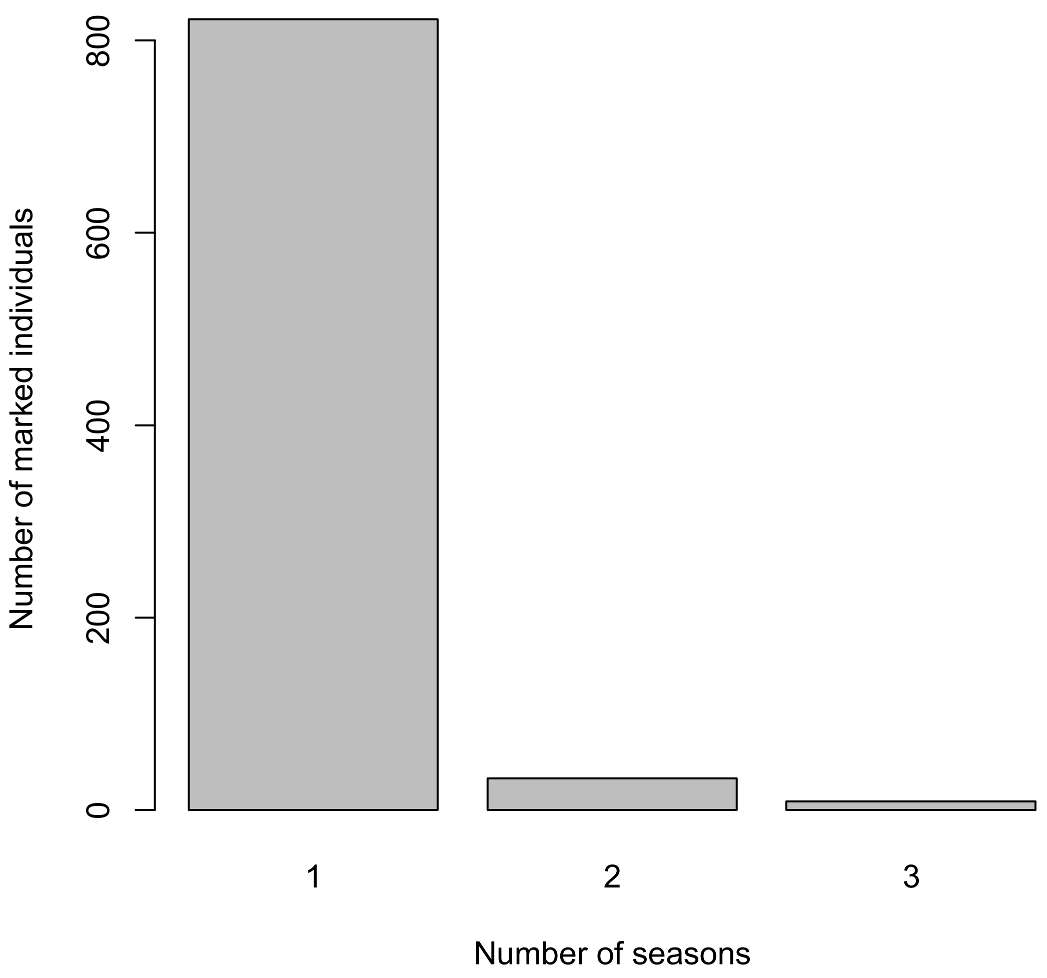

Number of marked individuals
0
200
400
600
800
1
2
3
Number of seasons
