# Supplementary material for: Abundance-based detectability in a spatially-explicit metapopulation: a case study on a vulnerable beetle species in hollow trees
Source: Oecologia. 2018 Jul 31;188(3):671–82. doi: 10.1007/s00442-018-4220-5 (PMC6208700; doi:10.1007/s00442-018-4220-5)
Supplement: Supplementary file 2 — Supplementary material 2 (PDF 269 kb) [file 442_2018_4220_MOESM2_ESM.pdf]

Online Resource 2: Modeling the number of observed individuals: rationale, calibration and prediction

F. Laroche, H. Paltto, T. Ranius

Contents

Rationale of the geometric sample model **1**

Building the response variable: positive count data **2**

Building covariates to explain positive count data **3**
Temporal covariates . . . 3
Hollow covariates . . . 6
Other tree features . . . 10
Girth . . . 11
Living status . . . 12
Sun exposure . . . 13

Fitting the geometric observation model to count data **14**
Downward model selection . . . 14
Mc Fadden R^2 . . . 17
Goodness of fit . . . 17

Predicting detectability for all surveys **18**

Predicting capacity parameters (K_i) for all trees **18**

Rationale of the geometric sample model

During a survey event, observation on a tree went on as long as new individuals were detected, without a priori absolute time limit for sampling. We modeled this as follows : each individual is detected at a rate ρ, and when no individual is detected during some time span t_{max}, sampling stops.

If one calls n_{tot} the true number of individuals in the tree when the observation takes place, then the time before detecting the first individual T_1 is exponentially distributed with rate $r_1 = n_{tot}\rho$. The time between detecting the first and the second individual is exponentially distributed with rate $r_2 = (n_{tot} - 1)\rho$. More generally, the time between detecting the kth and the $k + 1^{th}$ individual is exponentially distributed with rate $r_k = (n_{tot} - k + 1)\rho$.

Consequently, no individual is detected if $T_1 > t_{max}$. One individual is detected if $T_1 < t_{max}$ and $T_2 > t_{max}$. More generally, k individuals are detected if $T_1, ..., T_k < t_{max}$ and $T_{k+1} > t_{max}$. We now

derive the probability P_k that k individuals are detected, that is the probability that $T_1, ..., T_k < t_{max}$ and $T_{k+1} > t_{max}$. Because the times T_k are independent one from another:

$$P_k = e^{-t_{max} r_{k+1}} \Pi_{l=1}^{k} (1 - e^{-t_{max} r_l}) = e^{-t_{max} \rho (n_{tot} - k)} \Pi_{l=1}^{k} (1 - e^{-t_{max} \rho (n_{tot} - l + 1)})$$

This observation model is rather uneasy to adjust to data. However, one may consider the reasonable limit case where the individual detection rate ρ is low ($\rho \rightarrow 0$), the true number of individuals in the metapopulation is large ($n_{tot} \rightarrow +\infty$) and the product of both quantities $n_{tot}\rho$, which is the rate of detection if the first individual in the tree r_1, remains finite. Then P_k boils down to the classical geometric distribution with parameter $g = e^{-t_{max} r_1}$:

$$P_k = e^{-t_{max} r_1} (1 - e^{-t_{max} r_1})^k = g(1 - g)^k$$

We modeled the relationship between parameter g, temporal features of survey and tree features in a rather phenomenological way, using a logit link function and a linear combination of dependent variables (equation 1 of main text). Detectability at visit scale, i.e. the probability of detecting at least one individual, thus verifies $\phi = 1 - g$, which explains equation (2) of main text.

Building the response variable: positive count data

The number of oak trees in the Bjarka Saby area was 338. The number of surveys in the study area was 4151. Most of the surveys did not lead to any detection of individuals. The number of surveys with no individual observed was 3636.

The calibration of the geometric observation model (equation 1 in main text) relies on the surveys that yielded strictly positive numbers of observed individuals. The number of surveys with detection of at least one individuals is 515, over 145 trees. Figure S2.1 shows the distribution of the number of observed individuals among surveys which yielded detection of individuals.

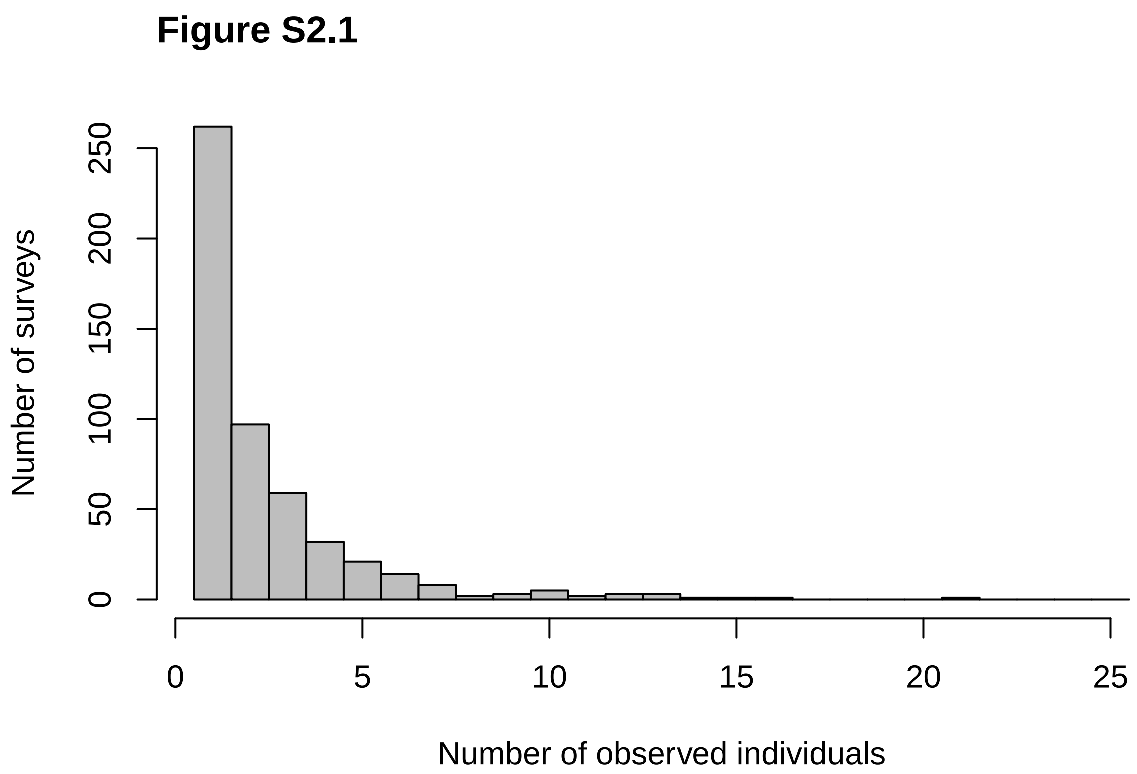

Figure S2.1 Histogram of the number of individuals observed in surveys where at least one individual is detected.

Building covariates to explain positive count data

Temporal covariates

We used three temporal covariates to explain the variation in the number of observed individuals presented above: date, time and temperature. We call them "temporal" because they can vary between two surveys performed on the same tree. The date is expressed in number of days since the 1st of January. Note that we pooled all the years together (thus neglecting any year effect). The time is expressed in number of minutes since previous noon. The temperature is expressed in Celsius degrees.

The time of survey depends on the date (Figure SX.2; mostly because night fall time depends on the period of the year).

Figure S2.2

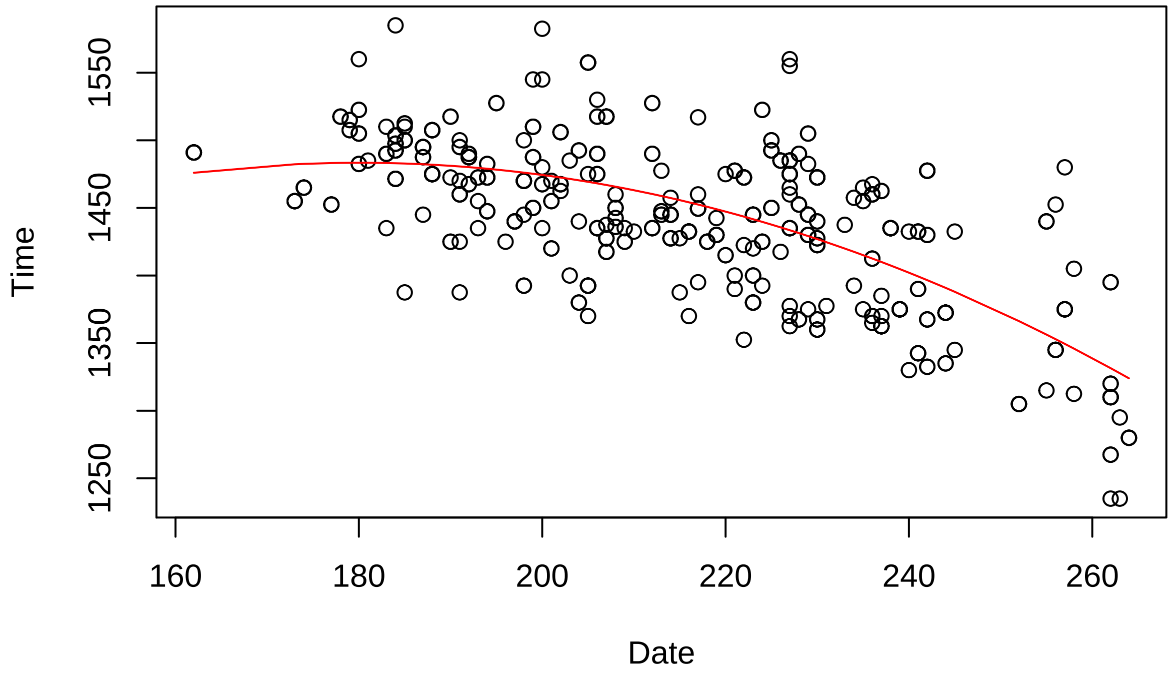

Figure S2.2 Relationship between time and date of survey in the T. opacus dataset *The red line indicates the prediction of our quadratic model.*

More precisely, the fit of a quadratic relationship between time and surveys yielded:

```
##
## Call:
## lm(formula = tim ~ dat + I(dat^2))
##
## Residuals:
##     Min      1Q  Median      3Q     Max
## -99.291 -29.065  -4.436  27.588 130.560
##
## Coefficients:
##                Estimate Std. Error t value Pr(>|t|)
## (Intercept) 750.226368 140.218589   5.350 1.33e-07 ***
## dat           8.144029   1.307378   6.229 9.84e-10 ***
## I(dat^2)     -0.022617   0.003024  -7.479 3.31e-13 ***
## ---
## Signif. codes:  0 '***' 0.001 '**' 0.01 '*' 0.05 '.' 0.1 ' ' 1
##
## Residual standard error: 40.97 on 508 degrees of freedom
##   (4 observations deleted due to missingness)
## Multiple R-squared:  0.4844, Adjusted R-squared:  0.4823
```

```
## F-statistic: 238.6 on 2 and 508 DF,  p-value: < 2.2e-16
```

It comes as no surprise that this relationship is modal with maximum (i.e. latest surveys in the night) around 180, which corresponds to June-July and the longest days of the year. We call “time” in subsequent analysis the residuals obtained from this model. By doing so, our “time” variable actually measures a time while controlling for the date.

We also found that temperature depends on both date and (residual) time (Figure SX.3), which we characterized with the quadratic model :

```
##
## Call:
## lm(formula = temp ~ dat + I(dat^2) + timRes)
##
## Residuals:
##     Min      1Q  Median      3Q     Max
## -8.1403 -1.6332  0.1353  1.3726  5.2598
##
## Coefficients:
##               Estimate Std. Error t value Pr(>|t|)
## (Intercept) -4.662e+01  7.726e+00  -6.034 3.13e-09 ***
## dat          5.896e-01  7.199e-02   8.190 2.25e-15 ***
## I(dat^2)    -1.391e-03  1.664e-04  -8.359 6.45e-16 ***
## timRes      -5.206e-03  2.499e-03  -2.083   0.0378 *
## ---
## Signif. codes:  0 '***' 0.001 '**' 0.01 '*' 0.05 '.' 0.1 ' ' 1
##
## Residual standard error: 2.229 on 495 degrees of freedom
##   (16 observations deleted due to missingness)
## Multiple R-squared:  0.1394, Adjusted R-squared:  0.1341
## F-statistic: 26.72 on 3 and 495 DF,  p-value: 4.973e-16
```

Figure S2.3

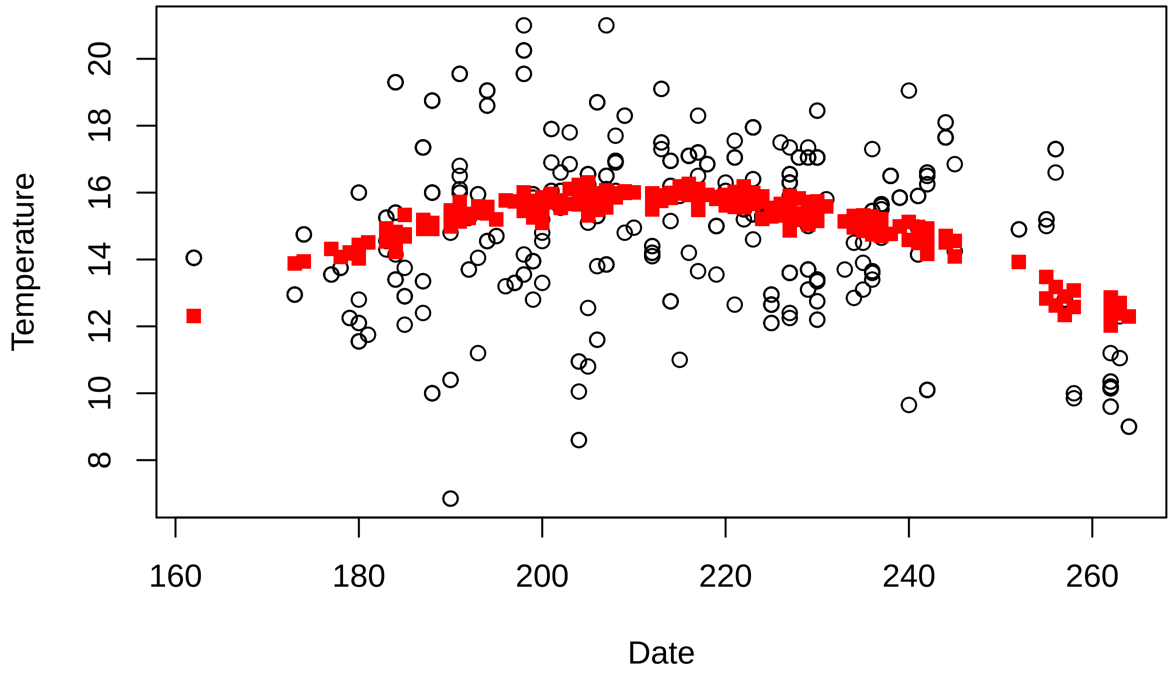

Figure S2.3 Relationship between temperature and date of survey in the T. opacus dataset *Red squares are fitted values of our quadratic model, time covariate is not presented.*

Here again results are rather intuitive. We find a modal relationship of temperature with respect to date, with maximum around 210, which corresponds to July-August. We find a negative effect of residual time: the later in the night, the colder. We call "temperature" in subsequent analysis the residuals obtained from this model. By doing so, our "temperature" variable actually measures a temperature while controlling for both date and time.

Hollow covariates

Hollows are described using two variables: (i) the area of the entrance (approximated using height x breadth, expressed in squared centimeters) and (ii) height of the entrance on the trunk (expressed in meters). Trees in the area of study could harbour several hollows (Figure S2.4).

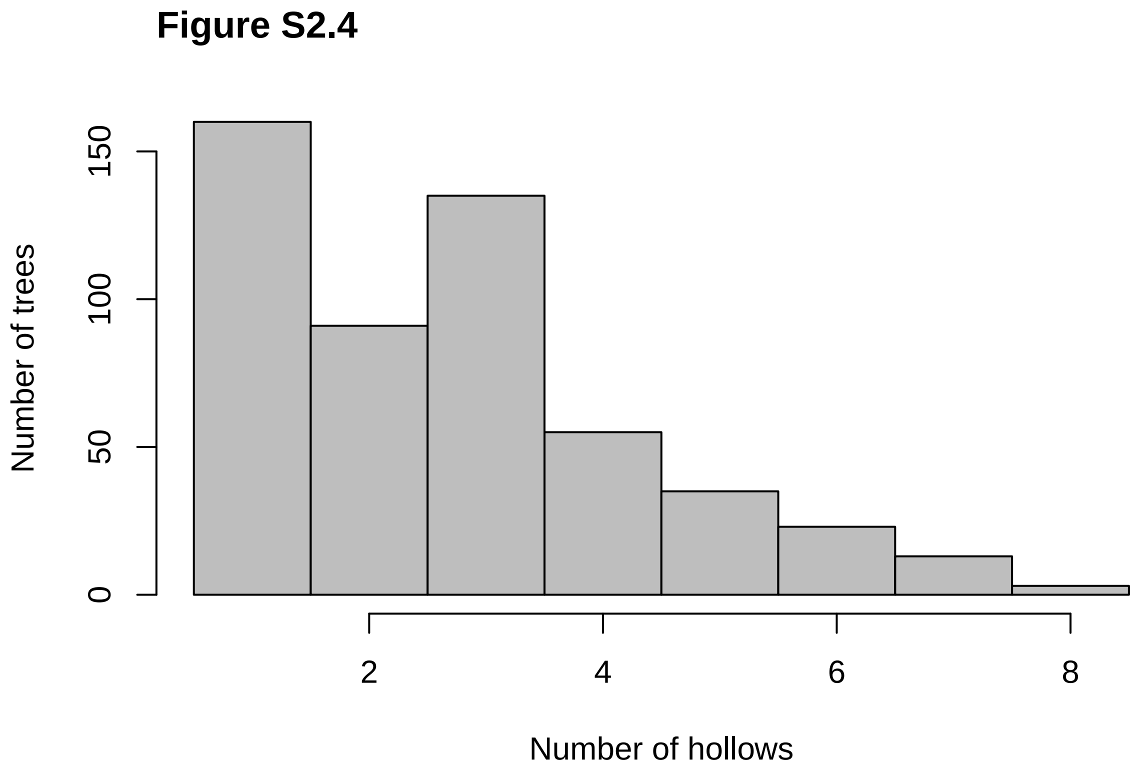

Figure S2.4 Histogram of the number of hollows per tree.

Hollows belonging to the same tree are aggregated together, considering the total area (summing across hollows) and the mean height. We thus obtain two covariates depicting hollow features at the tree level: the “mean hollow height” (Figure S2.5) and the “total hollow area” (Figure S2.6).

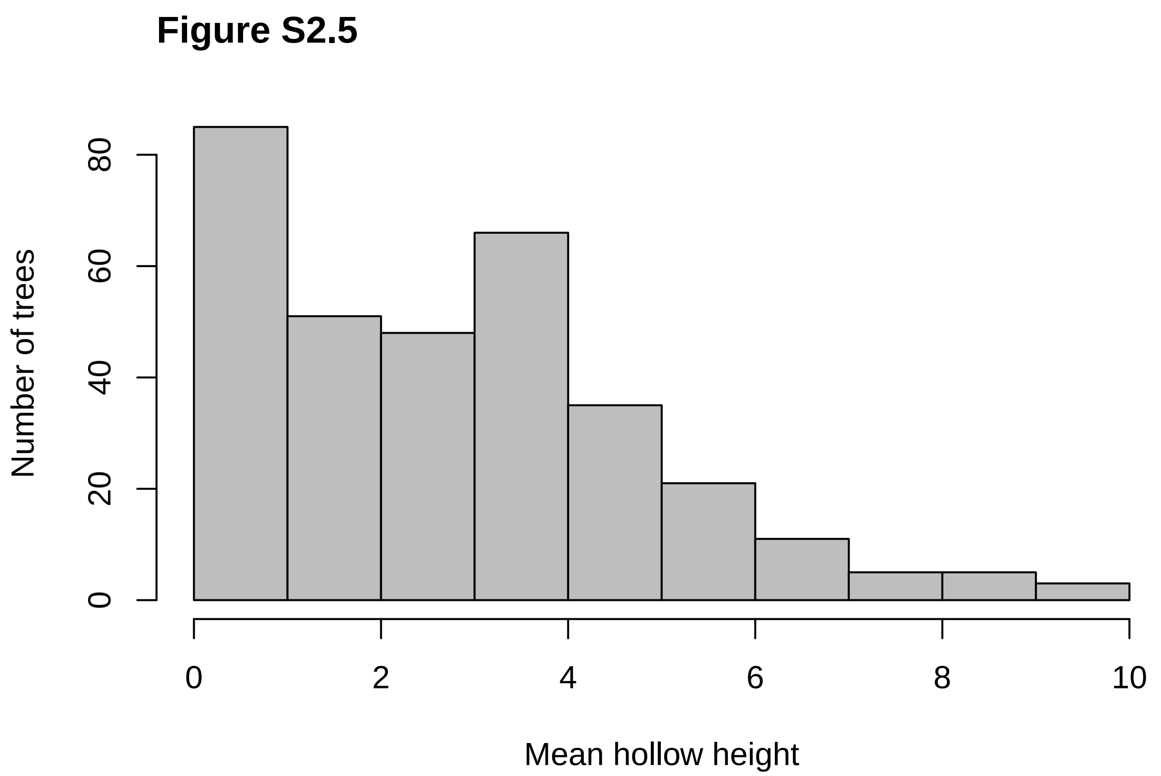

Figure S2.5 Histogram of the mean hollow height in trees. *Note that data was missing to compute the mean hollow height for 8 trees.*

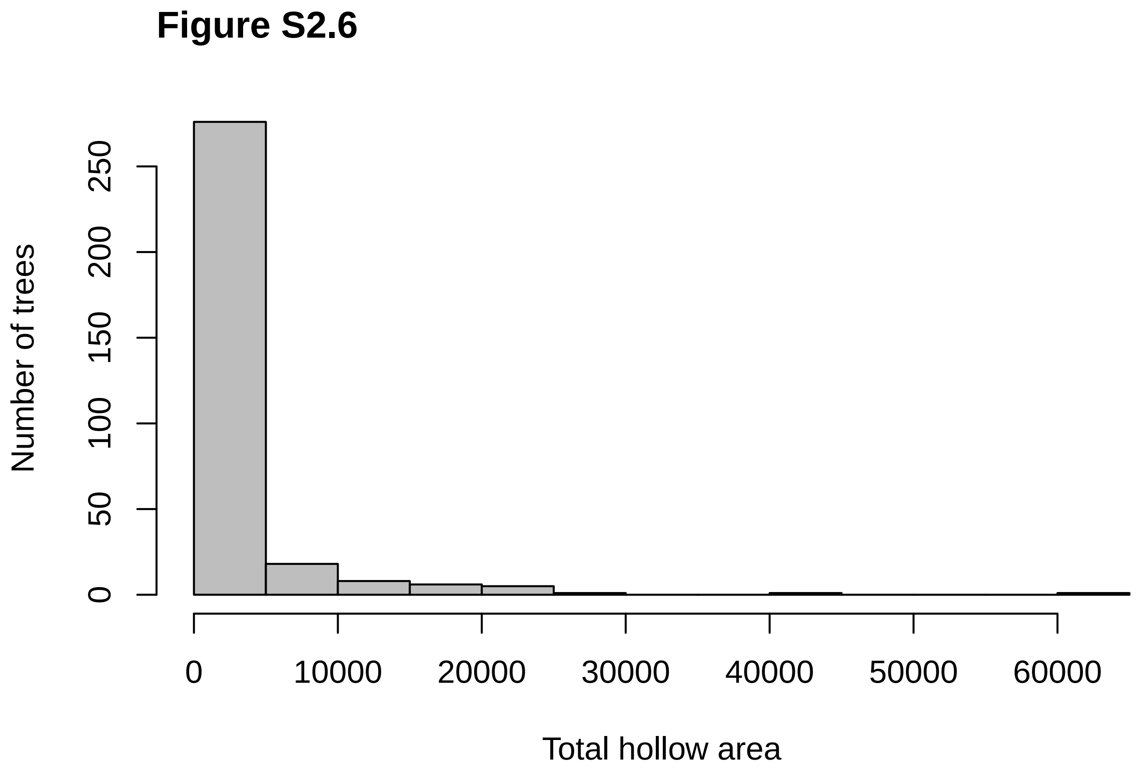

Figure S2.6 Histogram of the total hollow area per tree. *Note that data was missing to compute the total hollow area for 22 trees.*

The histogram of the total hollow area in trees is very skewed (Figure S2.6), which would potentially have generated statistical issues in subsequent analyses. We therefore log-transformed the total hollow area (Figure S2.7). From now, we call "Total hollow area" in subsequent analysis this log transformed variable.

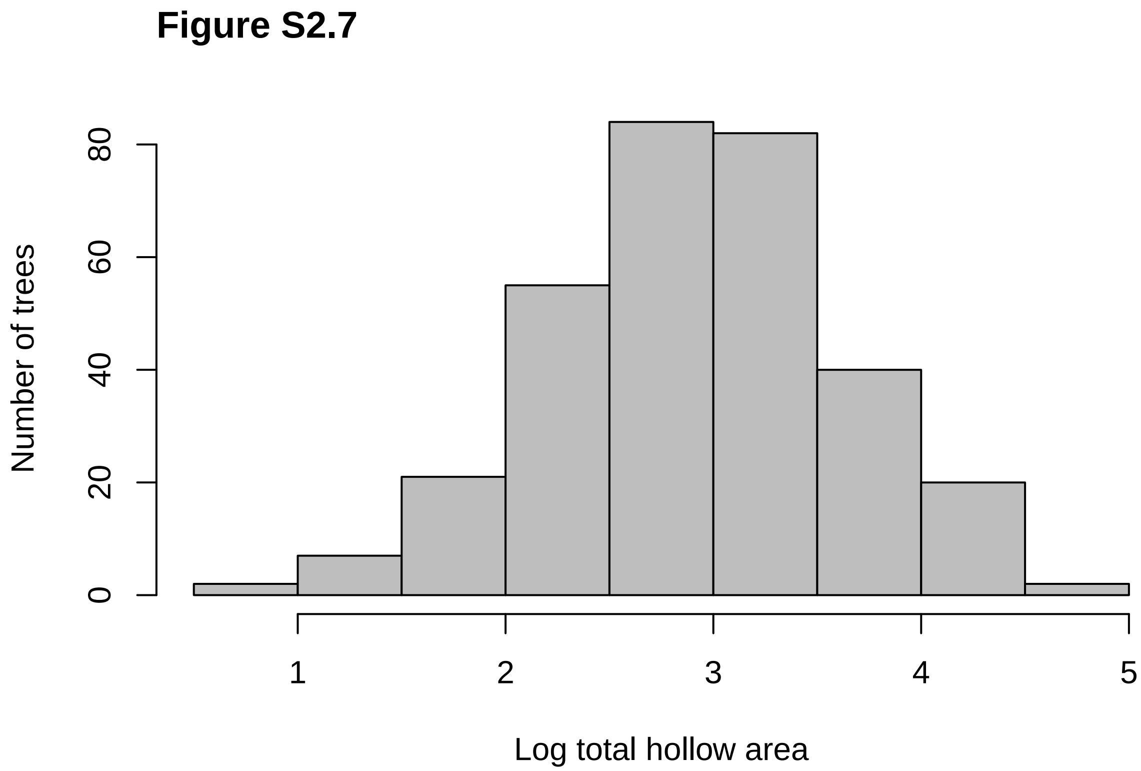

Figure S2.7 Histogram of the log-total hollow area per tree

In particular, there was no significant correlation between the total hollow area and the mean hollow height according to a Kendall correlation test:

```
##
##  Kendall's rank correlation tau
##
## data:  mHH and sHA
## z = 0.23056, p-value = 0.8177
## alternative hypothesis: true tau is not equal to 0
## sample estimates:
##        tau
## 0.00902062
```

Other tree features

In addition to hollow features mentioned above, we also included tree girth (in centimeters), tree living status (alive=1, dead=0) and sun exposure (qualitative assessment from 0 to 2).

Girth

Tree girth was related to mean hollow height and total hole area (Figure S2.8) as shown by the following statistical model:

```
##
## Call:
## lm(formula = girthTree ~ meanHolHeightTree + holAreaTreeFit)
##
## Residuals:
##     Min      1Q  Median      3Q     Max
## -245.79  -74.51  -16.56   60.06  302.21
##
## Coefficients:
##                   Estimate Std. Error t value Pr(>|t|)
## (Intercept)        241.363     25.184   9.584  < 2e-16 ***
## meanHolHeightTree    9.326      2.552   3.654 0.000304 ***
## holAreaTreeFit      40.326      7.977   5.055 7.38e-07 ***
## ---
## Signif. codes:  0 '***' 0.001 '**' 0.01 '*' 0.05 '.' 0.1 ' ' 1
##
## Residual standard error: 99.85 on 308 degrees of freedom
##   (27 observations deleted due to missingness)
## Multiple R-squared:  0.1113, Adjusted R-squared:  0.1055
## F-statistic: 19.28 on 2 and 308 DF,  p-value: 1.288e-08
```

These results are not surprising, they basically mean that bigger trees tend to harbour bigger holes, which stems from both a geometric constraint and an age effect of the tree. :

Figure S2.8

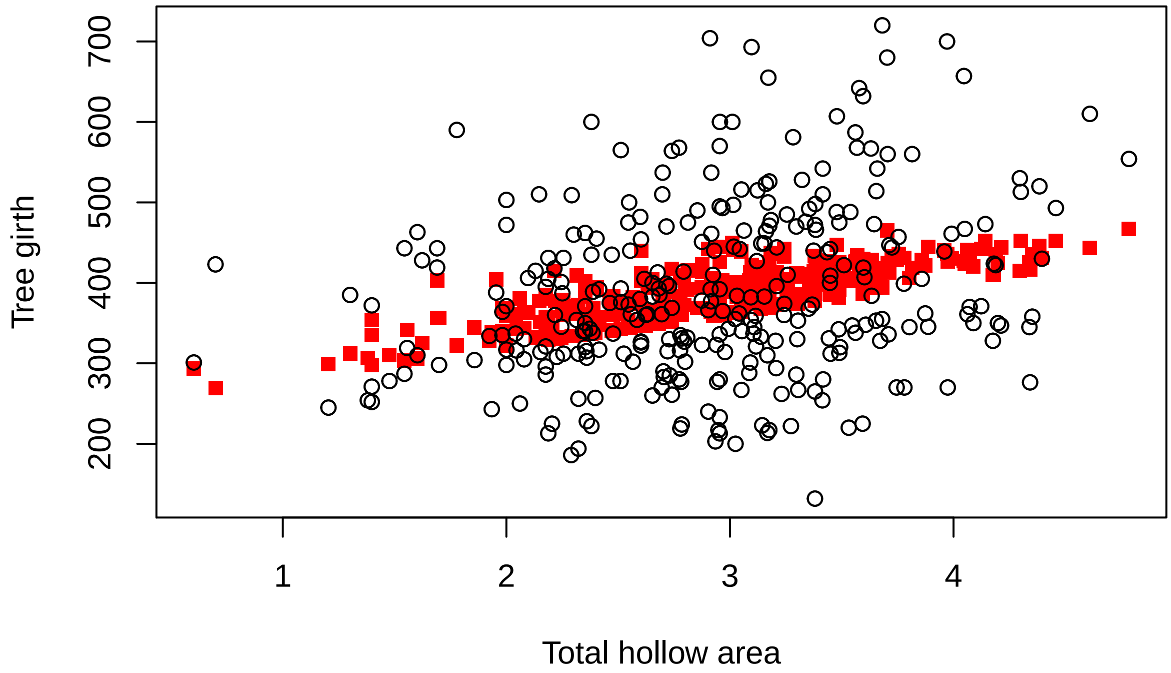

Figure S2.8 Relationship between tree girth and total hollow area *Red squares present the fit of the model. Note that girth measure was missing for the 2 trees in our dataset.*

This rather tight relationship may generate issues when using both girth and hollow features in the modeling of numbers of observed individuals. We therefore choose to keep only the residuals of the model presented above as our "girth" variable, that is the part of tree girth that is independent from hollow features. It may be seen as a measure of tree girth while controlling for the hollow features.

Living status

We did the same kind of analysis for living status as for girth, exploring potential relationships with hollow features through the following generalized linear model:

```
##
## Call:
## glm(formula = livingTree ~ meanHolHeightTree + holAreaTreeFit,
##     family = binomial)
##
## Deviance Residuals:
##     Min       1Q   Median       3Q      Max
## -2.2906   0.4124   0.5671   0.6909   1.0970
##
## Coefficients:
```

```
##                    Estimate Std. Error z value Pr(>|z|)
## (Intercept)         2.94306    0.70053   4.201 2.65e-05 ***
## meanHolHeightTree  0.15407    0.07455   2.067  0.03877 *
## holAreaTreeFit    -0.64027    0.21447  -2.985  0.00283 **
## ---
## Signif. codes:  0 '***' 0.001 '**' 0.01 '*' 0.05 '.' 0.1 ' ' 1
##
## (Dispersion parameter for binomial family taken to be 1)
##
##     Null deviance: 305.48  on 311  degrees of freedom
## Residual deviance: 291.49  on 309  degrees of freedom
##   (26 observations deleted due to missingness)
## AIC: 297.49
##
## Number of Fisher Scoring iterations: 4
```

We found that trees where holes were larger and closer to the ground were more likely to be dead. Here again we focused on the residual effect of the living status when controling for hollow features by considering the working residuals of this model as our living status variable.

Sun exposure

Sun exposure did not show any strong relationship with the hollow features, as shown by the linear model:

```
##
## Call:
## lm(formula = sunExpTree ~ meanHolHeightTree + holAreaTreeFit)
##
## Residuals:
##      Min       1Q   Median       3Q      Max
## -1.05471 -0.89195  0.02527  0.55037  1.20537
##
## Coefficients:
##                    Estimate Std. Error t value Pr(>|t|)
## (Intercept)         0.92296    0.18643   4.951 1.22e-06 ***
## meanHolHeightTree -0.01796    0.01892  -0.949    0.343
## holAreaTreeFit     0.03034    0.05898   0.514    0.607
## ---
## Signif. codes:  0 '***' 0.001 '**' 0.01 '*' 0.05 '.' 0.1 ' ' 1
##
## Residual standard error: 0.7381 on 308 degrees of freedom
##   (27 observations deleted due to missingness)
## Multiple R-squared:  0.003806,   Adjusted R-squared:  -0.002663
## F-statistic: 0.5883 on 2 and 308 DF,  p-value: 0.5559
```

Consequently, we kept it unchanged in the analysis.

Globally, after the treatments detailed above, the variables associated to hollow and tree features

did not show any strong correlation across trees :

```
##                    girthTreeRes   sunExpTree livingTreeRes   holAreaTree
## girthTreeRes       1.0000000000 -0.168003521   0.145819405  0.0004712215
## sunExpTree        -0.1680035214  1.000000000  -0.004669445  0.0291763603
## livingTreeRes      0.1458194053 -0.004669445   1.000000000 -0.0024582042
## holAreaTree        0.0004712215  0.029176360  -0.002458204  1.0000000000
## meanHolHeightTree  0.0018912027 -0.053519767  -0.008367959 -0.0108169988
##                   meanHolHeightTree
## girthTreeRes            0.001891203
## sunExpTree             -0.053519767
## livingTreeRes          -0.008367959
## holAreaTree            -0.010816999
## meanHolHeightTree       1.000000000
```

Fitting the geometric observation model to count data

Downward model selection

We performed downward model selection on a geometric generalized linear model of positive count data using date (quadratic), time (residual), temperature (residual), total hollow area, mean hollow height, tree girth (residual), tree living status (residual) and tree sun exposure. At each step, we identified the non-significant variable (p>0.05) with highest p-value (according to a χ^2 log-likelihood ratio test) and remove it from the model. We stopped when all the remaining variables were significant (p<0.05)

```
## [1] "STEP 1"
```

```
## Single term deletions
##
## Model:
## vecNObs ~ livingRes + girthRes + sunExp + meanHolHeight + holArea +
##     dat + dat2 + timRes + tempRes
##               Df Deviance    AIC     LRT  Pr(>Chi)
## <none>             1475.7 1495.7
## livingRes      1   1491.9 1509.9 16.1544 5.838e-05 ***
## girthRes       1   1482.8 1500.8  7.0340 0.0079976 **
## sunExp         1   1475.8 1493.8  0.0716 0.7890390
## meanHolHeight  1   1490.5 1508.5 14.8075 0.0001191 ***
## holArea        1   1492.4 1510.4 16.6949 4.390e-05 ***
## dat            1   1476.5 1494.5  0.7544 0.3850767
## dat2           1   1476.3 1494.3  0.6210 0.4306719
## timRes         1   1486.6 1504.6 10.9217 0.0009504 ***
## tempRes        1   1478.4 1496.4  2.6420 0.1040715
## ---
## Signif. codes:  0 '***' 0.001 '**' 0.01 '*' 0.05 '.' 0.1 ' ' 1
```

```
## [1] "STEP 2"
```

```
## Single term deletions
##
## Model:
## vecNObs ~ livingRes + girthRes + meanHolHeight + holArea + dat +
##     dat2 + timRes + tempRes
##               Df Deviance    AIC     LRT  Pr(>Chi)
## <none>             1475.8 1493.8
## livingRes      1   1491.9 1507.9 16.1275 5.922e-05 ***
## girthRes       1   1484.3 1500.3  8.5092 0.0035335 **
## meanHolHeight  1   1490.5 1506.5 14.7365 0.0001236 ***
## holArea        1   1493.1 1509.1 17.3534 3.103e-05 ***
## dat            1   1476.6 1492.6  0.7651 0.3817320
## dat2           1   1476.4 1492.4  0.6317 0.4267482
## timRes         1   1487.0 1503.0 11.1654 0.0008334 ***
## tempRes        1   1478.5 1494.5  2.6912 0.1009030
## ---
## Signif. codes:  0 '***' 0.001 '**' 0.01 '*' 0.05 '.' 0.1 ' ' 1
```

```
## [1] "STEP 3"
```

```
## Single term deletions
##
## Model:
## vecNObs ~ livingRes + girthRes + meanHolHeight + holArea + dat +
##     timRes + tempRes
##               Df Deviance    AIC     LRT  Pr(>Chi)
## <none>             1476.4 1492.4
## livingRes      1   1493.0 1507.0 16.5785 4.668e-05 ***
## girthRes       1   1484.9 1498.9  8.5191 0.0035144 **
## meanHolHeight  1   1490.9 1504.9 14.4653 0.0001428 ***
## holArea        1   1493.5 1507.5 17.0290 3.681e-05 ***
## dat            1   1478.3 1492.3  1.8889 0.1693306
## timRes         1   1487.3 1501.3 10.8692 0.0009778 ***
## tempRes        1   1479.4 1493.4  2.9522 0.0857602 .
## ---
## Signif. codes:  0 '***' 0.001 '**' 0.01 '*' 0.05 '.' 0.1 ' ' 1
```

```
## [1] "STEP 4"
```

```
## Single term deletions
##
## Model:
## vecNObs ~ livingRes + girthRes + meanHolHeight + holArea + timRes +
##     tempRes
##               Df Deviance    AIC     LRT  Pr(>Chi)
## <none>             1478.3 1492.3
## livingRes      1   1495.4 1507.4 17.0697 3.603e-05 ***
## girthRes       1   1486.1 1498.1  7.7870 0.0052622 **
## meanHolHeight  1   1492.7 1504.7 14.3821 0.0001492 ***
## holArea        1   1495.2 1507.2 16.8701 4.003e-05 ***
```

```
## timRes         1   1489.7 1501.7 11.3475 0.0007555 ***
## tempRes        1   1480.6 1492.6  2.3196 0.1277532
## ---
## Signif. codes:  0 '***' 0.001 '**' 0.01 '*' 0.05 '.' 0.1 ' ' 1
```

```
## [1] "STEP 5"
```

```
## Single term deletions
##
## Model:
## vecNObs ~ livingRes + girthRes + meanHolHeight + holArea + timRes
##               Df Deviance    AIC     LRT  Pr(>Chi)
## <none>              1480.6 1492.6
## livingRes      1   1497.3 1507.3 16.6957 4.388e-05 ***
## girthRes       1   1490.1 1500.1  9.4791 0.0020783 **
## meanHolHeight  1   1495.3 1505.3 14.6615 0.0001286 ***
## holArea        1   1498.7 1508.7 18.0350 2.169e-05 ***
## timRes         1   1492.0 1502.0 11.3886 0.0007389 ***
## ---
## Signif. codes:  0 '***' 0.001 '**' 0.01 '*' 0.05 '.' 0.1 ' ' 1
```

We thus retained the following geometric model:

```
##
## Call:
## glm(formula = vecNObs ~ livingRes + girthRes + meanHolHeight +
##     holArea + timRes, family = binomial, data = datDup)
##
## Deviance Residuals:
##     Min       1Q   Median       3Q      Max
## -1.5997  -0.9639  -0.8206   1.2184   2.0547
##
## Coefficients:
##                Estimate Std. Error z value Pr(>|z|)
## (Intercept)    0.656921   0.309623   2.122 0.033865 *
## livingRes     -0.742706   0.182656  -4.066 4.78e-05 ***
## girthRes      -0.001801   0.000588  -3.064 0.002184 **
## meanHolHeight  0.139169   0.036605   3.802 0.000144 ***
## holArea       -0.380096   0.090713  -4.190 2.79e-05 ***
## timRes        -0.005236   0.001564  -3.347 0.000817 ***
## ---
## Signif. codes:  0 '***' 0.001 '**' 0.01 '*' 0.05 '.' 0.1 ' ' 1
##
## (Dispersion parameter for binomial family taken to be 1)
##
##     Null deviance: 1554.9  on 1155  degrees of freedom
## Residual deviance: 1480.6  on 1150  degrees of freedom
## AIC: 1492.6
##
## Number of Fisher Scoring iterations: 4
```

Mc Fadden R^2

We computed the log-likelihood of the positive count data under a full geometric model (one parameter per observation) considering only surveys with no missing data in covariates, which equaled -466.7989054. The likelihood of the selected geometric model equaled -740.3154274, which corresponds to a deviance of 547.033044 when compared to the full geometric model. The null geometric model (one parameter only, used for all the surveys) yielded the log-likelihood -777.4303024, which corresponds to a deviance of 621.262794. We could therefore compute the Mac Fadden R^2 of the model (defined as $1 - ModelDeviance/NullDeviance$), which equaled 0.119482.

Goodness of fit

We explored the goodness of fit of the selected geometric model by simulating 1000 virtual datasets using maximum likelihood parameter estimates provided above. For each simulated dataset, we computed the new maximum likelihood estimates, and reported corresponding log-likelihood (Figure S2.9). We observed that likelihood of real data did not significantly differ from that of simulations, suggesting an adequate fit of the model to the positive count data.

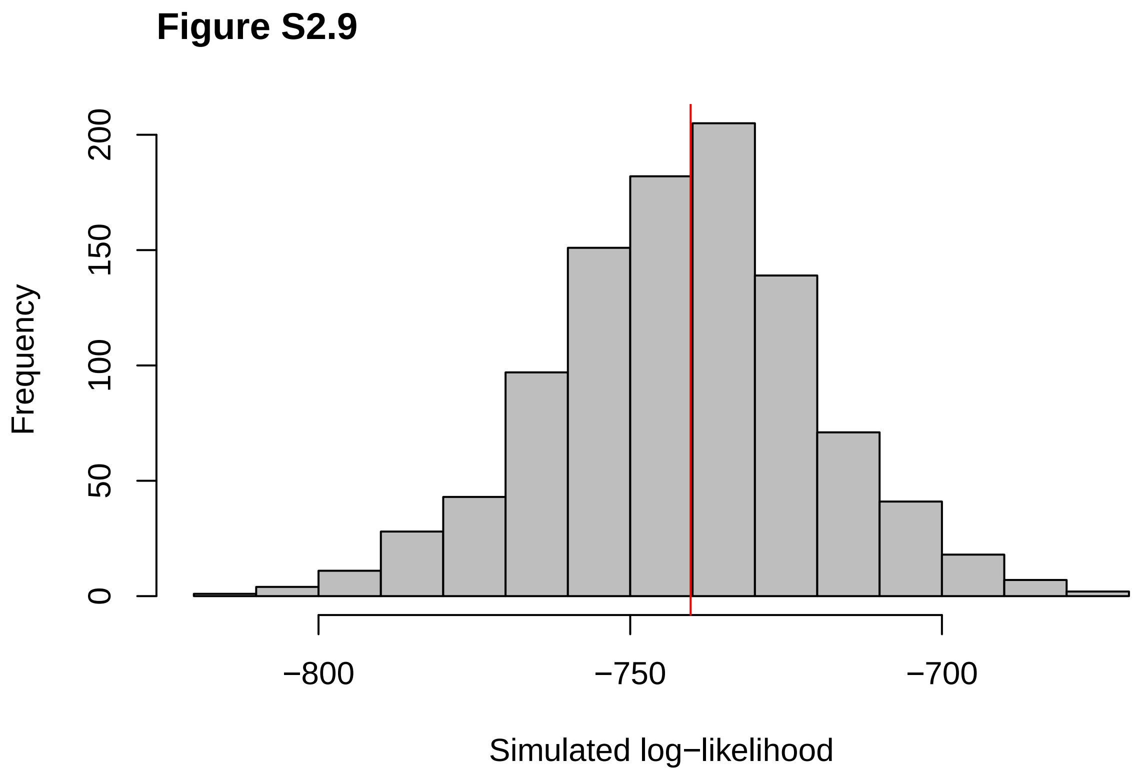

Figure S2.9 Histogram of simulated log-likelihood under the selected geometric observation model *The vertical red bar indicates the maximum likelihood of the real data.*

Predicting detectability for all surveys

We derived the prediction of our selected model for all the surveys of our dataset (whether individuals were detected or not). When data was missing for either mean hollow height, total hollow area or tree girth (see figure legends above for numbers of trees involved), we filled the data set using the average across all the trees where data was available. Similarly, the number of surveys where time was missing equaled 8. We filled these missing (residual) time data using the average across all surveys where the residual time was available. We could then predict the parameter g of the geometric distribution for each tree and each survey, even those without observed individuals (Figure S2.9), and the corresponding distribution of detectability ($\phi = 1 - g$) at survey scale is provided in Figure 1A of main text.

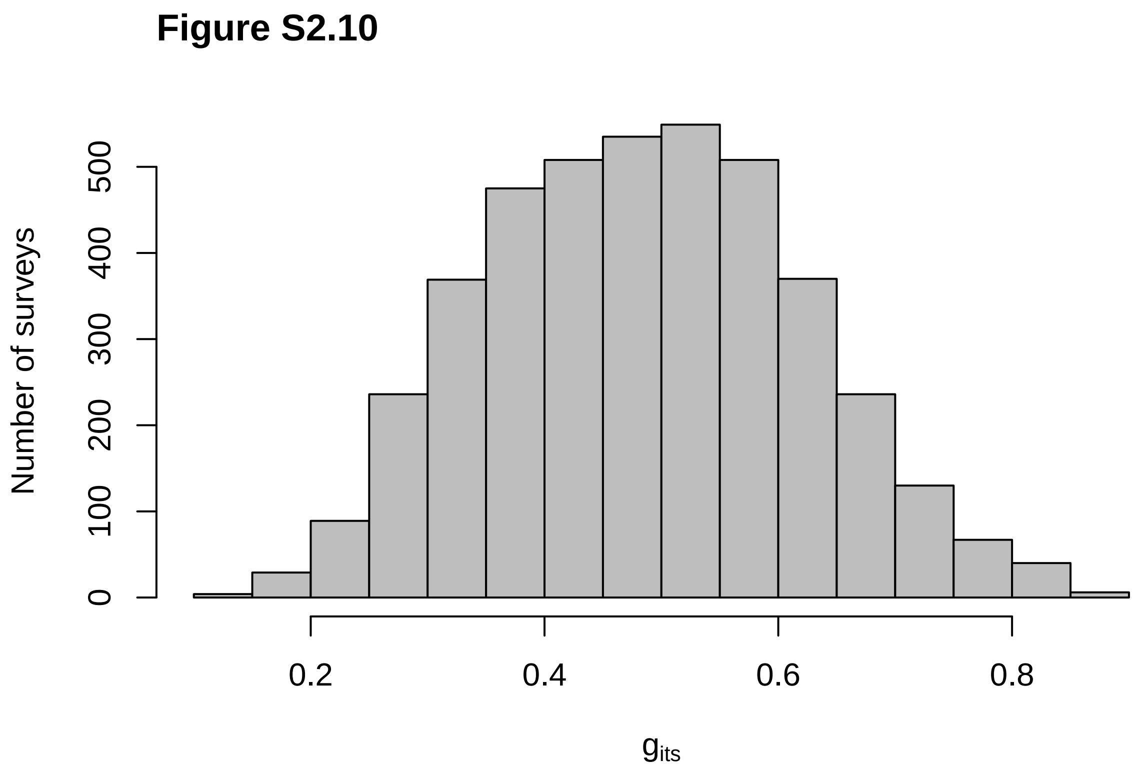

Figure S2.10 Distribution of predicted g parameter over all the surveys of the T. opacus dataset

Predicting capacity parameters (K_i) for all trees

We derived the K_i parameters by simply deriving our selected geometric model prediction for each tree while artificially putting the (residual) time covariate at 0 (Figure S2.10). This provided us with g_i, which we could transform to K_i using equation 7 of main text.

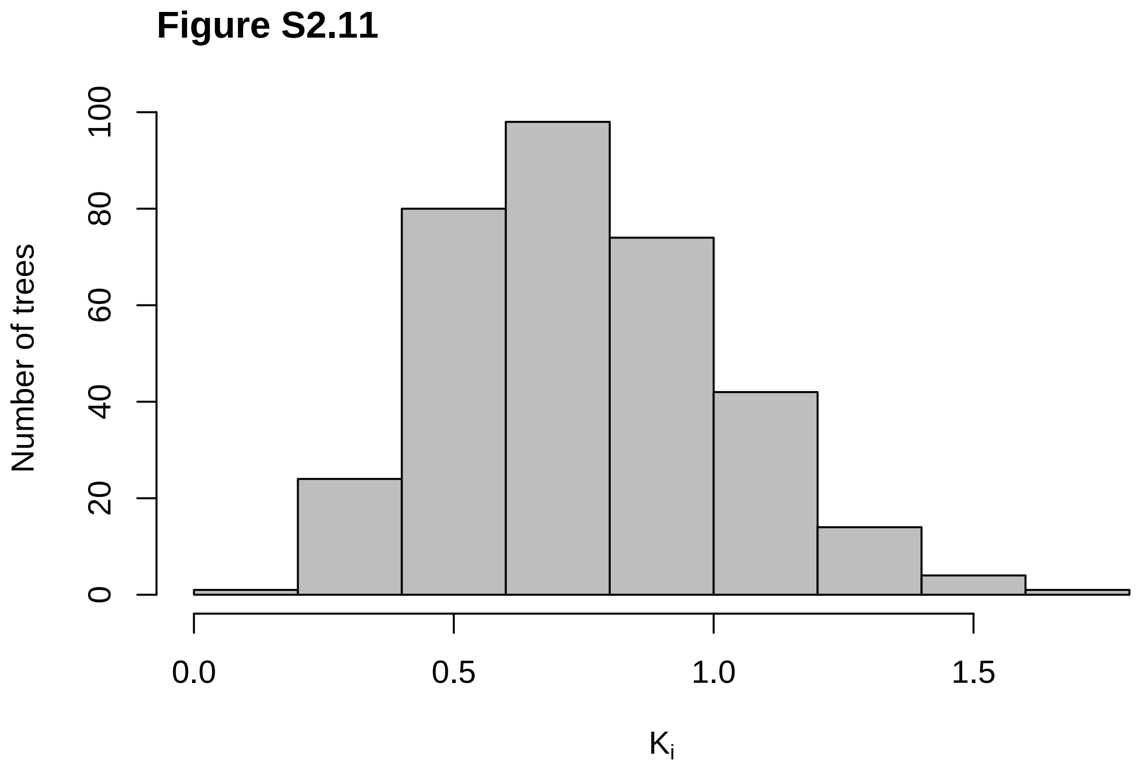

Figure S2.11 Distribution of tree carrying capacities
