# Supplementary material for: Abundance-based detectability in a spatially-explicit metapopulation: a case study on a vulnerable beetle species in hollow trees
Source: Oecologia. 2018 Jul 31;188(3):671–82. doi: 10.1007/s00442-018-4220-5 (PMC6208700; doi:10.1007/s00442-018-4220-5)
Supplement: Supplementary file 3 — Supplementary material 3 (PDF 106 kb) [file 442_2018_4220_MOESM3_ESM.pdf]

Online Resource 3: Comparing occupancy-based and abundance-based detectability at year scale

F. Laroche, H. Paltto, T. Ranius

In this appendix, we computed a classic detectability estimate based on repeated presence/absence observation the same year in the same tree, and we compared it to our detectability estimate based on numbers of individuals detected when detection occurs. Importantly, these two estimates did not use the same data. The occupancy-based detectability estimate used presence/absence in all the surveys done the same year in the same tree while the abundance-based estimate only considered the surveys where individuals are detected, and used numbers of individuals. In particular, the former is not influenced by numbers of individuals during detection, while the latter is not influenced by the number of unsuccessful surveys.

We performed the comparison between the two detectability estimates using models of detectability with parameter per tree × *year* combination. We considered a tree i and a year j. Using notations similar to main text, we call s_{ij} the number of surveys done at this tree × year combination. We further call k_{ij} the number of surveys where *T. opacus* was detected and a_{ij} the total number of individuals which were detected across all successful surveys at this tree × year combination. Recall that ϕ_{ij} is the detectability of the *T. opacus* at visit scale for this tree × year combination. Then, in the occupancy-based framework, the likelihood of ϕ_{ij} only depends on observations through s_{ij} and k_{ij}, and verifies:

$$L_{oc}(\phi_{ij}; s_{ij}, k_{ij}) = p_{ij}\phi_{ij}^{k_{ij}}(1-\phi_{ij})^{s_{ij}-k_{ij}} + (1-p_{ij})I_{k_{ij}=0}$$

In the abundance-based framework, the likelihood of ϕ_{ij} only depends on observations through k_{ij} and a_{ij}, and verifies:

$$L_{ab}(\phi_{ij}; k_{ij}, a_{ij}) = p_{ij}\phi_{ij}^{a_{ij}-k_{ij}}(1-\phi_{ij})^{k_{ij}} + (1-p_{ij})I_{k_{ij}=0}$$

We adopted a bayesian perspective upon detectability estimation when comparing occupancy-based and abundance-based frameworks. We assumed that ϕ_{ij} prior distribution is uniform over $[0,1]$. Then the *a posteriori* density of ϕ_{ij} in the occupancy-based (denoted $d_{oc}(\phi_{ij}; s_{ij}, k_{ij})$) and the abundance-based framework (denoted $d_{ab}(\phi_{ij}; k_{ij}, a_{ij})$) respectively verify:

$$d_{oc}(\phi_{ij}; s_{ij}, k_{ij}) = \frac{p_{ij}\phi_{ij}^{k_{ij}}(1-\phi_{ij})^{s_{ij}-k_{ij}} + (1-p_{ij})I_{k_{ij}=0}}{p_{ij}B(k_{ij}+1, s_{ij}-k_{ij}+1) + (1-p_{ij})I_{k_{ij}=0}}$$

and

$$d_{ab}(\phi_{ij}; k_{ij}, a_{ij}) = \frac{p_{ij}\phi_{ij}^{a_{ij}-k_{ij}}(1-\phi_{ij})^{k_{ij}} + (1-p_{ij})I_{k_{ij}=0}}{p_{ij}B(a_{ij}-k_{ij}+1, k_{ij}+1) + (1-p_{ij})I_{k_{ij}=0}}$$

respectively.

When comparing detectability estimates, we only considered tree × year configuration where a *T. opacus* population was detected at least once (i.e. $k_{ij} > 0$) (this represents 251 tree × year configuration). In these particular cases, *a posteriori* distributions of ϕ_{ij} in the occupancy-based and the abundance-based frameworks reduced to beta distributions with parameters $(\alpha_{oc}, \beta_{oc}) = (k_{ij} + 1, s_{ij} - k_{ij} + 1)$ and $(\alpha_{ab}, \beta_{ab}) = (a_{ij} - k_{ij} + 1, k_{ij} + 1)$ respectively.

We used the expectation of a posteriori distribution as an estimate of detectability in both abundance-based and occupancy-based frameworks. These estimates verify:

$$\hat{\phi}_{ij,ab} = \frac{a_{ij} - k_{ij} + 1}{a_{ij} + 2} \tag{1}$$

and

$$\hat{\phi}_{ij,oc} = \frac{k_{ij} + 1}{s_{ij} + 2} \tag{2}$$

We plotted these estimates for our 251 tree × year configurations in Figure 2 of main text. The correlation between the two kinds of estimate equaled 0.5396282, which was strongly significant based on a Pearson correlation test:

```
##
##  Pearson's product-moment correlation
##
## data:  vecMeanPAPos and vecMeanPOPos
## t = 10.114, df = 249, p-value < 2.2e-16
## alternative hypothesis: true correlation is not equal to 0
## 95 percent confidence interval:
##  0.4455811 0.6218947
## sample estimates:
##       cor
## 0.5396282
```

To evaluate whether the size of this correlation could be deemed strong, we simulated 10^{4} virtual datasets of observation upon the 251 tree × year configurations. In all the simulations, we kept the same number of surveys as in the original dataset. We generated number of observed individuals in each survey using geometric sampling and taking the parameter equal to the abundance-based estimate in the real dataset. When some sampling yielded no observed individual over all the surveys of some tree × year configuration, we did the sampling again, since all the tree × year configurations have at least one individual detected in one survey in the real dataset. For each simulated dataset, we obtained abundance-based and occupancy-based detectability estimates using formulas (1) and (2) above and computed the Pearson correlation across the 251 tree × year configurations. Simulated datasets exactly abide by our model hypothesis that number of observed individuals is geometrically distributed. Then, in these examples, we know for sure that abundance-based and occupancy-based estimates do estimate the same quantity.

We observed that the correlation between these two estimates obtained from real data was among the highest possible values observed from simulated datasets (Figure S3.1). This suggests that the correlation observed from real data is a strong effect.

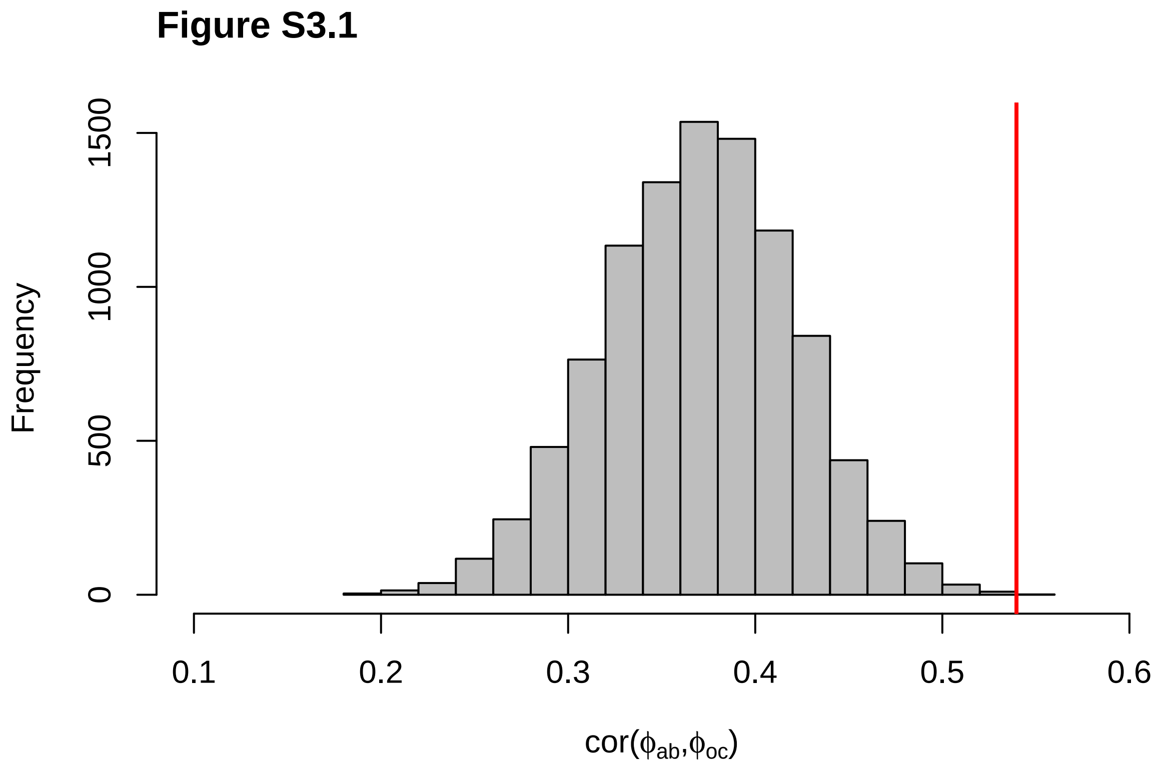

FigureS3.1 Observed and simulated correlations between occupancy-based and abundance-based estimates. *The histogram indicates the distribution of the 10^{4} correlation coefficients obtained from simulated datasets. The vertical red bar indicates the position of the correlation between estimates observed in the real dataset.*
