# Supplementary material for: Abundance-based detectability in a spatially-explicit metapopulation: a case study on a vulnerable beetle species in hollow trees
Source: Oecologia. 2018 Jul 31;188(3):671–82. doi: 10.1007/s00442-018-4220-5 (PMC6208700; doi:10.1007/s00442-018-4220-5)
Supplement: Supplementary file 4 — Supplementary material 4 (PDF 191 kb) [file 442_2018_4220_MOESM4_ESM.pdf]

Online Resource 4: Deriving the pseudo-likelihood of occupancy data and controlling the quality of metapopulation parameters estimation

F. Laroche, H. Paltto, T. Ranius

Contents

Deriving the pseudo-likelihood formula 1

Quadratic error over a wide range of parameters 3

Bias and variance/covariance at one parameter set 11

Summary of results regarding estimation quality 14

Deriving the pseudo-likelihood formula

We define o_{it} as the observed occupancy in tree i at year t; $o_{it} = 0$ means that no individual was observed in the visits performed in tree i at year t (including cases where no visit was done in tree i at year t) while $o_{it} = 1$ means that at least one visit in tree i at year t reported some individuals. The observed occupancies at year t were noted as $o_{.t} = (o_{1t}, o_{2t}, ..., o_{Nt})$. We use the same index notations for real occupancies z_{it}. We denote $o_{..}$ the total array of observed occupancies.

The likelihood of observed occupancies verifies:

$$\begin{aligned} P(o_{..}|\Theta) &= P(o_{.1}|\Theta) \prod_{t=2}^{6} P(o_{.t}| \bigcap_{s=1}^{t-1} o_{.s}, \Theta) \\ &= E\left[P(o_{.1}|z_{.1})|\Theta\right] \prod_{t=2}^{6} E\left[P(o_{.t}|z_{.t})| \bigcap_{s=1}^{t-1} o_{.s}, \Theta\right] \end{aligned} \tag{1}$$

Noting that $P(o_{.t}|z_{.t}) = \prod_{i=1}^{N} P(o_{it}|z_{it})$, (1) becomes:

$$P(o_{..}|\Theta) = E\left[\prod_{i=1}^{N} P(o_{i1}|z_{i1})|\Theta\right] \prod_{t=2}^{6} E\left[\prod_{i=1}^{N} P(o_{it}|z_{it})| \bigcap_{s=1}^{t-1} o_{.s}, \Theta\right] \tag{2}$$

We assume that, for all t, the z_{it}) random variables can be considered as probabilistically independent given past observations $\bigcap_{s=1}^{t-1} o_{.s}$. Note that this approximation is exact for $t > 1$ when observation is perfect. Our approximation implies that (2) becomes:

$$\begin{aligned} P(o_{..}|\Theta) &= \prod_{i=1}^{N} E\left[P(o_{i1}|z_{i1})|\Theta\right] \prod_{t=2}^{6} \prod_{i=1}^{N} E\left[P(o_{it}|z_{it})| \bigcap_{s=1}^{t-1} o_{.s}, \Theta\right] \\ &= \prod_{i=1}^{N} \left[p_{i1}\left[o_{i1}\Phi_{i1} + (1-o_{i1})(1-\Phi_{i1})\right] + (1-p_{i1})(1-o_{i1})\right] \\ &\quad \times \prod_{t=2}^{6} \prod_{i=1}^{N} \left[\left[o_{it}\Phi_{it} + (1-o_{it})(1-\Phi_{it})\right] + (1-p_{it})(1-o_{it})\right] \end{aligned} \tag{3}$$

where $p_{it} = P(z_{it} = 1 | \bigcap_{s=1}^{t-1} o_{.s}, \Theta)$. One can therefore compute the pseudolikelihood of data from $p_{..}$.

$p_{..}$ is computed as follows:

$$\begin{array}{rcl} p_{it} & = & P(z_{it} = 1 | \bigcap_{s=1}^{t-1} o_{.s}, \Theta) \\ & = & E[P(z_{it} = 1 | z_{.t-1}) | \bigcap_{s=1}^{t-1} o_{.s}, \Theta] \end{array} \tag{4}$$

Introducing equation (4) of main text into (4) here yields:

$$\begin{array}{rcl} p_{it} & = & E[\gamma_{i,t-1}(1 - z_{i,t-1}) + [1 - \epsilon_{i,t-1}(1 - \gamma_{i,t-1})] \, z_{i,t-1} | \bigcap_{s=1}^{t-1} o_{.s}, \Theta] \\ & = & E[(1 - e^{-c_{out}} \prod_{j=1, j\neq i}^{N} (1 - \rho_{i,j,t-1}))(1 - z_{i,t-1}) + (1 - \epsilon_{i,t-1} e^{-c_{out}} \prod_{j=1, j\neq i}^{N} (1 - \rho_{i,j,t-1})) z_{i,t-1} | \bigcap_{s=1}^{t-1} o_{.s}, \Theta] \\ & = & E[(1 - e^{-c_{out}} \prod_{j=1, j\neq i}^{N} exp(-z_{j,t-1} c K_j e^{-\alpha d_{ij}}))(1 - z_{i,t-1}) \\ & & +(1 - \epsilon_{i,t-1} e^{-c_{out}} \prod_{j=1, j\neq i}^{N} exp(-z_{j,t-1} c K_j e^{-\alpha d_{ij}})) z_{i,t-1} | \bigcap_{s=1}^{t-1} o_{.s}, \Theta] \\ & = & E[(1 - exp(-c_{out} - c \sum_{j=1, j\neq i}^{N} z_{j,t-1} K_j e^{-\alpha d_{ij}}))(1 - z_{i,t-1}) \\ & & +(1 - \epsilon_{i,t-1} exp(-c_{out} - c \sum_{j=1, j\neq i}^{N} z_{j,t-1} K_j e^{-\alpha d_{ij}})) z_{i,t-1} | \bigcap_{s=1}^{t-1} o_{.s}, \Theta] \\ & = & 1 - E[(1 - z_{i,t-1}(1 - \epsilon_{i,t-1})) exp(-c_{out} - c \sum_{j=1, j\neq i}^{N} z_{j,t-1} K_j e^{-\alpha d_{ij}}) | \bigcap_{s=1}^{t-1} o_{.s}, \Theta] \\ & = & 1 - e^{-c_{out}} E[(1 - z_{i,t-1}(1 - \epsilon_{i,t-1})) \prod_{j=1, j\neq i}^{N} exp(-c z_{j,t-1} K_j e^{-\alpha d_{ij}}) | \bigcap_{s=1}^{t-1} o_{.s}, \Theta] \end{array} \tag{5}$$

Our approximation implies that for all t, the z_{it} random variables can be considered as probabilistically independent given past and present observations $\bigcap_{s=1}^{t} o_{.s}$ (proof not shown). Thus (5) becomes:

$$\begin{array}{rcl} p_{it} & = & 1 - e^{-c_{out}} E\left[(1 - z_{i,t-1}(1 - \epsilon_{i,t-1})) | \bigcap_{s=1}^{t-1} o_{.s}, \Theta\right] \\ & & \times \prod_{j=1, j\neq i}^{N} E\left[exp(-c z_{j,t-1} K_j e^{-\alpha d_{ij}}) | \bigcap_{s=1}^{t-1} o_{.s}, \Theta\right] \\ & = & 1 - \left[1 - E\left[z_{i,t-1} | \bigcap_{s=1}^{t-1} o_{.s}, \Theta\right] (1 - \epsilon_{i,t-1})\right] \\ & & \times e^{-c_{out}} \prod_{j=1, j\neq i}^{N} E\left[exp(-c z_{j,t-1} K_j e^{-\alpha d_{ij}}) | \bigcap_{s=1}^{t-1} o_{.s}, \Theta\right] \end{array} \tag{6}$$

We introduce $q_{it} = E\left[z_{i,t} | \bigcap_{s=1}^{t} o_{.s}, \Theta\right]$. Then (6) becomes:

$$p_{it} = 1 - [1 - q_{i,t-1}(1 - \epsilon_{i,t-1})] \, e^{-c_{out}} \prod_{j=1, j\neq i}^{N} \left[1 - q_{i,t-1}(1 - exp(-c K_j e^{-\alpha d_{ij}}))\right] \tag{7}$$

We introduce $\tilde{\rho}_{i,j,t} = q_{j,t-1}(1 - exp(-c K_j e^{-\alpha d_{ij}}))$ into (7):

$$p_{it} = 1 - [1 - q_{i,t-1}(1 - \epsilon_{i,t-1})] \, e^{-c_{out}} \prod_{j=1, j\neq i}^{N} \left(1 - \tilde{\rho}_{i,j,t}\right) \tag{8}$$

We introduce $\tilde{\gamma}_{it} = 1 - e^{-c_{out}} \prod_{j=1, j\neq i}^{N} \left[1 - \tilde{\rho}_{i,j,t}\right]$ into (8):

$$\begin{array}{rcl} p_{it} & = & 1 - [1 - q_{i,t-1}(1 - \epsilon_{i,t-1})] \, (1 - \tilde{\gamma}_{it}) \\ & = & \tilde{\gamma}_{it} \, (1 - q_{i,t-1}) + q_{i,t-1}(1 - \epsilon_{i,t-1} \, (1 - \tilde{\gamma}_{it})) \end{array} \tag{9}$$

One can then compute $p_{.t}$ from $q_{.,t-1}$.

$q_{.,t}$ can be computed as follows, starting from its definition:

$$\begin{aligned} q_{it} &= E\left[z_{i,t} | \bigcap_{s=1}^{t} o_{.s}, \Theta\right] \\ &= P\left(z_{i,t} = 1 | \bigcap_{s=1}^{t} o_{.s}, \Theta\right) \end{aligned} \tag{10}$$

Applying Bayes theorem on (10) yields:

$$\begin{aligned} q_{it} &= \frac{P(o_{it}|z_{i,t}=1)P\left(z_{it}=1| \bigcap_{s=1}^{t-1} o_{.s},\Theta\right)}{P\left(o_{it}| \bigcap_{s=1}^{t-1} o_{.s},\Theta\right)} \\ &= \frac{(\Phi_{it}o_{it}+(1-\Phi_{it})(1-o_{it}))p_{it}}{(1-p_{it})P(o_{i,t}|z_{i,t}=0)+p_{it}P(o_{i,t}|z_{i,t}=1)} \\ &= \frac{p_{it}(\Phi_{it}o_{it}+(1-\Phi_{it})(1-o_{it}))}{(1-p_{it})(1-o_{i,t})+p_{it}(\Phi_{it}o_{it}+(1-\Phi_{it})(1-o_{it}))} \end{aligned} \tag{11}$$

(11) can be formulated more simply:

$$\begin{cases} q_{it} = \frac{p_{it}(1-\Phi_{it})}{(1-p_{it})+p_{it}(1-\Phi_{it})} & if\ o_{it} = 0 \\ q_{it} = 1 & if\ o_{it} = 1 \end{cases} \tag{12}$$

One can then compute $q_{.t}$ from $p_{.,t-1}$. Therefore $p_{..}$ and $q_{..}$ can be integrally determined from $p_{.1}$.

We determine $p_{.1}$ as a fixed point of the system of equations:

$$\forall 1 \leq i \leq N, p_i^\star = \gamma_i^\star\,(1 - p_i^\star) + p_i^\star(1 - \epsilon_i^\star\,(1 - \gamma_i^\star)) \tag{13}$$

where $\epsilon_i^\star = \epsilon_{it} = e^{-sK_i}$, $\gamma_i^\star = 1 - e^{-c_{out}} \prod_{j=1,j\neq i}^{N} \left[1 - \rho_{i,j}^\star\right]$ and $\rho_{i,j}^\star = p_j^\star(1 - exp(-cK_j e^{-\alpha d_{ij}}))$. This means that we assume that (i) the metapopulation follows a stationary dynamics in the area of study and that (ii) the distribution of occupancies under this stationary dynamics is well approximated by independent occupancies in each tree i with probability $p_i^\star$.

The $\gamma_i^\star$ are also reported as colonization probability of trees in main text.

Quadratic error over a wide range of parameters

In main text and Online Resource 2, we have explained how we obtained estimates of detectability and carrying capacity for each tree of the area of study. In the previous section, we provided a pseudo-likelihood for metapopulation parameters (c, s, α and c_{out}). We suggested to obtain estimates for these parameters by maximizing the pseudo-likelihood given observed occupancy data, detectability and carrying capacity estimates. In this appendix, we briefly explored the quadratic error of these estimates for the open spatially realistic metapopulation model (no parameter forced to 0). All the analyses were done using a log-scale for parameters (or equivalently considering log-parameters instead of parameters).

We used the following procedure: (i) we chose a set of metapopulation log-parameters ($log_{10}(c)$, $log_{10}(s)$, $log_{10}(\alpha)$ and $log_{10}(c_{out})$); (ii) we simulated 100 occupancy dataset by running the metapopulation model on our study landscape; (iii) on each simulated occupancy dataset, we applied our

detectability model to obtain a dataset of observed occupancies; (iv) on each dataset of observed occupancies, we estimated metapopulation parameters using our pseudo-likelihood and derived log-estimates; (iv) for each set of log-estimates, we computed the square euclidean distance to the real log-parameter set that generated the data (i.e. a multidimensional quadratic error); (v) we reported the average quadratic error over our 100 replicates. Note that steps (ii) and (iii) make use of estimated tree carrying capacities and detectability.

We applied this procedure to various sets of metapopulation parameters to get an overview of quadratic error associated to our estimation procedure. We generated log-parameters to be tested, using the log-parameters estimated from our real dataset ($log_{10}(c) =$ -0.9933023, $log_{10}(s) =$ -0.0744784, $log_{10}(\alpha) =$ 1.4181249, $log_{10}(c_{out}) =$ -0.8335349; see Table 4 of main text) as a reference.

We built two arrays of log-parameter sets for our trial: the first contained all the sets obtained by applying shifts of $\pm log_{10}(2)$ to the reference set of log-parameters; the second contained all the sets obtained by applying shifts of $\pm log_{10}(10)$ to the reference set of log-parameters. Each array thus contains 81 combinations (the reference log-parameter set included). In the natural scale of parameters, these two arrays correspond to multiplying/dividing parameters by 2 or 10 respectively.

We reported below the full results of our exploration of quadratic error (QE) for these two arrays of parameter sets.

```
## [1] "Quadratic error for log-parameter sets obtained through log(2) shifts:"

##          logC        logS     logA    logCOut        QE
## 1  -1.2943323 -0.37550835 1.117095 -1.1345649 0.9994632
## 2  -1.2943323 -0.37550835 1.117095 -0.8335349 0.4940545
## 3  -1.2943323 -0.37550835 1.117095 -0.5325049 0.5795762
## 4  -1.2943323 -0.37550835 1.418125 -1.1345649 2.2868142
## 5  -1.2943323 -0.37550835 1.418125 -0.8335349 2.2382815
## 6  -1.2943323 -0.37550835 1.418125 -0.5325049 2.1690323
## 7  -1.2943323 -0.37550835 1.719155 -1.1345649 3.3208083
## 8  -1.2943323 -0.37550835 1.719155 -0.8335349 3.0110975
## 9  -1.2943323 -0.37550835 1.719155 -0.5325049 2.9583664
## 10 -1.2943323 -0.07447835 1.117095 -1.1345649 0.4099700
## 11 -1.2943323 -0.07447835 1.117095 -0.8335349 0.4234091
## 12 -1.2943323 -0.07447835 1.117095 -0.5325049 0.4888089
## 13 -1.2943323 -0.07447835 1.418125 -1.1345649 1.4615469
## 14 -1.2943323 -0.07447835 1.418125 -0.8335349 1.8475538
## 15 -1.2943323 -0.07447835 1.418125 -0.5325049 1.2986479
## 16 -1.2943323 -0.07447835 1.719155 -1.1345649 2.6368988
## 17 -1.2943323 -0.07447835 1.719155 -0.8335349 2.8622339
## 18 -1.2943323 -0.07447835 1.719155 -0.5325049 2.8090244
## 19 -1.2943323  0.22655164 1.117095 -1.1345649 0.3366732
## 20 -1.2943323  0.22655164 1.117095 -0.8335349 0.4075572
## 21 -1.2943323  0.22655164 1.117095 -0.5325049 0.5158161
## 22 -1.2943323  0.22655164 1.418125 -1.1345649 0.9871625
## 23 -1.2943323  0.22655164 1.418125 -0.8335349 0.8004571
## 24 -1.2943323  0.22655164 1.418125 -0.5325049 1.2736062
## 25 -1.2943323  0.22655164 1.719155 -1.1345649 1.6259675
```

```
## 26 -1.2943323  0.22655164 1.719155 -0.8335349 2.3565777
## 27 -1.2943323  0.22655164 1.719155 -0.5325049 2.5943481
## 28 -0.9933023 -0.37550835 1.117095 -1.1345649 0.3019491
## 29 -0.9933023 -0.37550835 1.117095 -0.8335349 0.3215364
## 30 -0.9933023 -0.37550835 1.117095 -0.5325049 0.3097653
## 31 -0.9933023 -0.37550835 1.418125 -1.1345649 1.0826419
## 32 -0.9933023 -0.37550835 1.418125 -0.8335349 0.8771128
## 33 -0.9933023 -0.37550835 1.418125 -0.5325049 0.8192448
## 34 -0.9933023 -0.37550835 1.719155 -1.1345649 2.3576565
## 35 -0.9933023 -0.37550835 1.719155 -0.8335349 2.5543565
## 36 -0.9933023 -0.37550835 1.719155 -0.5325049 2.0158778
## 37 -0.9933023 -0.07447835 1.117095 -1.1345649 0.2497273
## 38 -0.9933023 -0.07447835 1.117095 -0.8335349 0.2897578
## 39 -0.9933023 -0.07447835 1.117095 -0.5325049 0.3066056
## 40 -0.9933023 -0.07447835 1.418125 -1.1345649 0.5314614
## 41 -0.9933023 -0.07447835 1.418125 -0.8335349 0.5061893
## 42 -0.9933023 -0.07447835 1.418125 -0.5325049 0.5532826
## 43 -0.9933023 -0.07447835 1.719155 -1.1345649 2.3360989
## 44 -0.9933023 -0.07447835 1.719155 -0.8335349 1.8798339
## 45 -0.9933023 -0.07447835 1.719155 -0.5325049 2.2182548
## 46 -0.9933023  0.22655164 1.117095 -1.1345649 0.2432085
## 47 -0.9933023  0.22655164 1.117095 -0.8335349 0.2987300
## 48 -0.9933023  0.22655164 1.117095 -0.5325049 0.3727861
## 49 -0.9933023  0.22655164 1.418125 -1.1345649 0.3589289
## 50 -0.9933023  0.22655164 1.418125 -0.8335349 0.4351741
## 51 -0.9933023  0.22655164 1.418125 -0.5325049 0.5160091
## 52 -0.9933023  0.22655164 1.719155 -1.1345649 0.8680860
## 53 -0.9933023  0.22655164 1.719155 -0.8335349 1.3411138
## 54 -0.9933023  0.22655164 1.719155 -0.5325049 1.8707182
## 55 -0.6922723 -0.37550835 1.117095 -1.1345649 0.2436850
## 56 -0.6922723 -0.37550835 1.117095 -0.8335349 0.2827395
## 57 -0.6922723 -0.37550835 1.117095 -0.5325049 0.6920330
## 58 -0.6922723 -0.37550835 1.418125 -1.1345649 0.3059031
## 59 -0.6922723 -0.37550835 1.418125 -0.8335349 0.3142938
## 60 -0.6922723 -0.37550835 1.418125 -0.5325049 0.3855344
## 61 -0.6922723 -0.37550835 1.719155 -1.1345649 1.2936728
## 62 -0.6922723 -0.37550835 1.719155 -0.8335349 1.1524352
## 63 -0.6922723 -0.37550835 1.719155 -0.5325049 1.3581269
## 64 -0.6922723 -0.07447835 1.117095 -1.1345649 0.2477820
## 65 -0.6922723 -0.07447835 1.117095 -0.8335349 0.2248129
## 66 -0.6922723 -0.07447835 1.117095 -0.5325049 0.2452397
## 67 -0.6922723 -0.07447835 1.418125 -1.1345649 0.2880458
## 68 -0.6922723 -0.07447835 1.418125 -0.8335349 0.3270684
## 69 -0.6922723 -0.07447835 1.418125 -0.5325049 0.3618935
## 70 -0.6922723 -0.07447835 1.719155 -1.1345649 0.6143708
## 71 -0.6922723 -0.07447835 1.719155 -0.8335349 0.7086242
## 72 -0.6922723 -0.07447835 1.719155 -0.5325049 0.8029367
## 73 -0.6922723  0.22655164 1.117095 -1.1345649 0.2807967
```

```
## 74 -0.6922723  0.22655164 1.117095 -0.8335349 0.3099480
## 75 -0.6922723  0.22655164 1.117095 -0.5325049 0.3174648
## 76 -0.6922723  0.22655164 1.418125 -1.1345649 0.3069057
## 77 -0.6922723  0.22655164 1.418125 -0.8335349 0.3158121
## 78 -0.6922723  0.22655164 1.418125 -0.5325049 0.4077612
## 79 -0.6922723  0.22655164 1.719155 -1.1345649 0.4205293
## 80 -0.6922723  0.22655164 1.719155 -0.8335349 0.4324744
## 81 -0.6922723  0.22655164 1.719155 -0.5325049 0.6019922
```

```
## [1] "Quadratic error for log-parameter sets obtained through log(10) shifts:"
```

```
##           logC        logS      logA    logCOut        QE
## 1  -1.993302342 -1.07447835 0.4181249 -1.8335349 5.2097737
## 2  -1.993302342 -1.07447835 0.4181249 -0.8335349 2.8205132
## 3  -1.993302342 -1.07447835 0.4181249  0.1664651 3.4342395
## 4  -1.993302342 -1.07447835 1.4181249 -1.8335349 5.5366931
## 5  -1.993302342 -1.07447835 1.4181249 -0.8335349 4.2700006
## 6  -1.993302342 -1.07447835 1.4181249  0.1664651 4.2276844
## 7  -1.993302342 -1.07447835 2.4181249 -1.8335349 5.1167020
## 8  -1.993302342 -1.07447835 2.4181249 -0.8335349 4.2245148
## 9  -1.993302342 -1.07447835 2.4181249  0.1664651 4.3697945
## 10 -1.993302342 -0.07447835 0.4181249 -1.8335349 3.1597701
## 11 -1.993302342 -0.07447835 0.4181249 -0.8335349 1.2055264
## 12 -1.993302342 -0.07447835 0.4181249  0.1664651 2.8966746
## 13 -1.993302342 -0.07447835 1.4181249 -1.8335349 4.1102972
## 14 -1.993302342 -0.07447835 1.4181249 -0.8335349 3.0190384
## 15 -1.993302342 -0.07447835 1.4181249  0.1664651 3.4884235
## 16 -1.993302342 -0.07447835 2.4181249 -1.8335349 3.8308261
## 17 -1.993302342 -0.07447835 2.4181249 -0.8335349 3.8460170
## 18 -1.993302342 -0.07447835 2.4181249  0.1664651 4.2910753
## 19 -1.993302342  0.92552165 0.4181249 -1.8335349 4.1986637
## 20 -1.993302342  0.92552165 0.4181249 -0.8335349 4.0800561
## 21 -1.993302342  0.92552165 0.4181249  0.1664651 4.9275911
## 22 -1.993302342  0.92552165 1.4181249 -1.8335349 1.8352526
## 23 -1.993302342  0.92552165 1.4181249 -0.8335349 3.2009464
## 24 -1.993302342  0.92552165 1.4181249  0.1664651 5.3304976
## 25 -1.993302342  0.92552165 2.4181249 -1.8335349 3.8564140
## 26 -1.993302342  0.92552165 2.4181249 -0.8335349 4.1692698
## 27 -1.993302342  0.92552165 2.4181249  0.1664651 5.4011649
## 28 -0.993302342 -1.07447835 0.4181249 -1.8335349 5.2315351
## 29 -0.993302342 -1.07447835 0.4181249 -0.8335349 3.8782939
## 30 -0.993302342 -1.07447835 0.4181249  0.1664651 4.1112827
## 31 -0.993302342 -1.07447835 1.4181249 -1.8335349 4.9640856
## 32 -0.993302342 -1.07447835 1.4181249 -0.8335349 2.1755781
## 33 -0.993302342 -1.07447835 1.4181249  0.1664651 3.7957224
## 34 -0.993302342 -1.07447835 2.4181249 -1.8335349 5.6943531
## 35 -0.993302342 -1.07447835 2.4181249 -0.8335349 4.7016358
## 36 -0.993302342 -1.07447835 2.4181249  0.1664651 4.3378675
## 37 -0.993302342 -0.07447835 0.4181249 -1.8335349 4.8969160
```

```
## 38 -0.993302342 -0.07447835 0.4181249 -0.8335349 3.6232082
## 39 -0.993302342 -0.07447835 0.4181249  0.1664651 4.2051525
## 40 -0.993302342 -0.07447835 1.4181249 -1.8335349 0.9098862
## 41 -0.993302342 -0.07447835 1.4181249 -0.8335349 0.5178842
## 42 -0.993302342 -0.07447835 1.4181249  0.1664651 2.1189931
## 43 -0.993302342 -0.07447835 2.4181249 -1.8335349 4.5714426
## 44 -0.993302342 -0.07447835 2.4181249 -0.8335349 4.0292424
## 45 -0.993302342 -0.07447835 2.4181249  0.1664651 4.3377989
## 46 -0.993302342  0.92552165 0.4181249 -1.8335349 3.9794277
## 47 -0.993302342  0.92552165 0.4181249 -0.8335349 4.4036122
## 48 -0.993302342  0.92552165 0.4181249  0.1664651 4.9701159
## 49 -0.993302342  0.92552165 1.4181249 -1.8335349 2.2699980
## 50 -0.993302342  0.92552165 1.4181249 -0.8335349 3.7222287
## 51 -0.993302342  0.92552165 1.4181249  0.1664651 5.1081921
## 52 -0.993302342  0.92552165 2.4181249 -1.8335349 1.9444280
## 53 -0.993302342  0.92552165 2.4181249 -0.8335349 3.7234398
## 54 -0.993302342  0.92552165 2.4181249  0.1664651 5.2191893
## 55  0.006697658 -1.07447835 0.4181249 -1.8335349 5.1720101
## 56  0.006697658 -1.07447835 0.4181249 -0.8335349 5.4294549
## 57  0.006697658 -1.07447835 0.4181249  0.1664651 4.5574940
## 58  0.006697658 -1.07447835 1.4181249 -1.8335349 3.1793861
## 59  0.006697658 -1.07447835 1.4181249 -0.8335349 3.0156944
## 60  0.006697658 -1.07447835 1.4181249  0.1664651 3.8234401
## 61  0.006697658 -1.07447835 2.4181249 -1.8335349 5.9196714
## 62  0.006697658 -1.07447835 2.4181249 -0.8335349 3.2621567
## 63  0.006697658 -1.07447835 2.4181249  0.1664651 4.0188195
## 64  0.006697658 -0.07447835 0.4181249 -1.8335349 5.3726154
## 65  0.006697658 -0.07447835 0.4181249 -0.8335349 5.3557276
## 66  0.006697658 -0.07447835 0.4181249  0.1664651 4.3060562
## 67  0.006697658 -0.07447835 1.4181249 -1.8335349 1.9160076
## 68  0.006697658 -0.07447835 1.4181249 -0.8335349 0.2858416
## 69  0.006697658 -0.07447835 1.4181249  0.1664651 2.0641115
## 70  0.006697658 -0.07447835 2.4181249 -1.8335349 2.5390030
## 71  0.006697658 -0.07447835 2.4181249 -0.8335349 1.5222652
## 72  0.006697658 -0.07447835 2.4181249  0.1664651 3.6613040
## 73  0.006697658  0.92552165 0.4181249 -1.8335349 3.2607770
## 74  0.006697658  0.92552165 0.4181249 -0.8335349 3.9556713
## 75  0.006697658  0.92552165 0.4181249  0.1664651 4.2659428
## 76  0.006697658  0.92552165 1.4181249 -1.8335349 4.3669636
## 77  0.006697658  0.92552165 1.4181249 -0.8335349 5.0040241
## 78  0.006697658  0.92552165 1.4181249  0.1664651 4.7406640
## 79  0.006697658  0.92552165 2.4181249 -1.8335349 1.5926089
## 80  0.006697658  0.92552165 2.4181249 -0.8335349 3.1036273
## 81  0.006697658  0.92552165 2.4181249  0.1664651 4.5562130
```

We analyzed these results in several steps. First, we explored whether the QE differed between the two datasets (Figure S4.1). We observed a marked difference, with parameter sets obtained through $log_{10}(10)$ shifts yielding much higher quadratic errors of estimation than thus obtained

from $log_{10}(2)$.

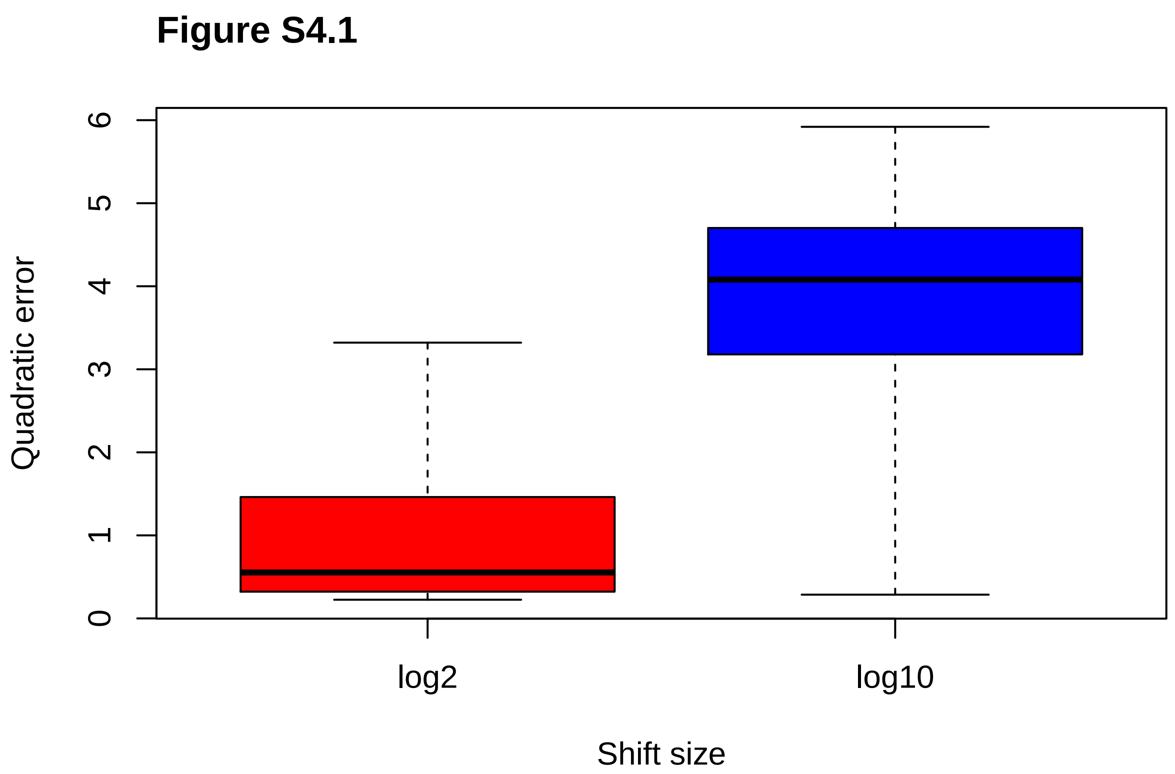

Figure S4.1: Quadratic errors in the two arrays of parameters. *The horizontal thick bar is the median, boxes encompass 75% of the data and whiskers cover 100% of the data. Color code red and blue for log-parameters obtained through $log_{10}(2)$ and $log_{10}(10)$ shifts respectively are consistently used across the figures.*

This difference in QE between the two arrays is readily explained when considering the true occupancy datasets (obtained at step [ii] of our testing procedure depicted above) that are at the basis of estimation. Indeed, log-parameter sets obtained through $log_{10}(10)$ shifts generate either a very high or a very low proportion of occupied sites over the 6 years of study (Figure S4.2). This means that the signal used for estimation is saturating. In such zones of the parameter space, differences among parameter sets do not generate difference on realized occupancy datasets, thus rendering accurate identification of parameters impossible. For instance, if the metapopulation is full at all time in the observed dataset for some survival parameter value, then any larger value of survival parameter will be equally likely and accurate estimate of the true value of survival parameter will be impossible. This explains why the QE for these parameter sets is high.

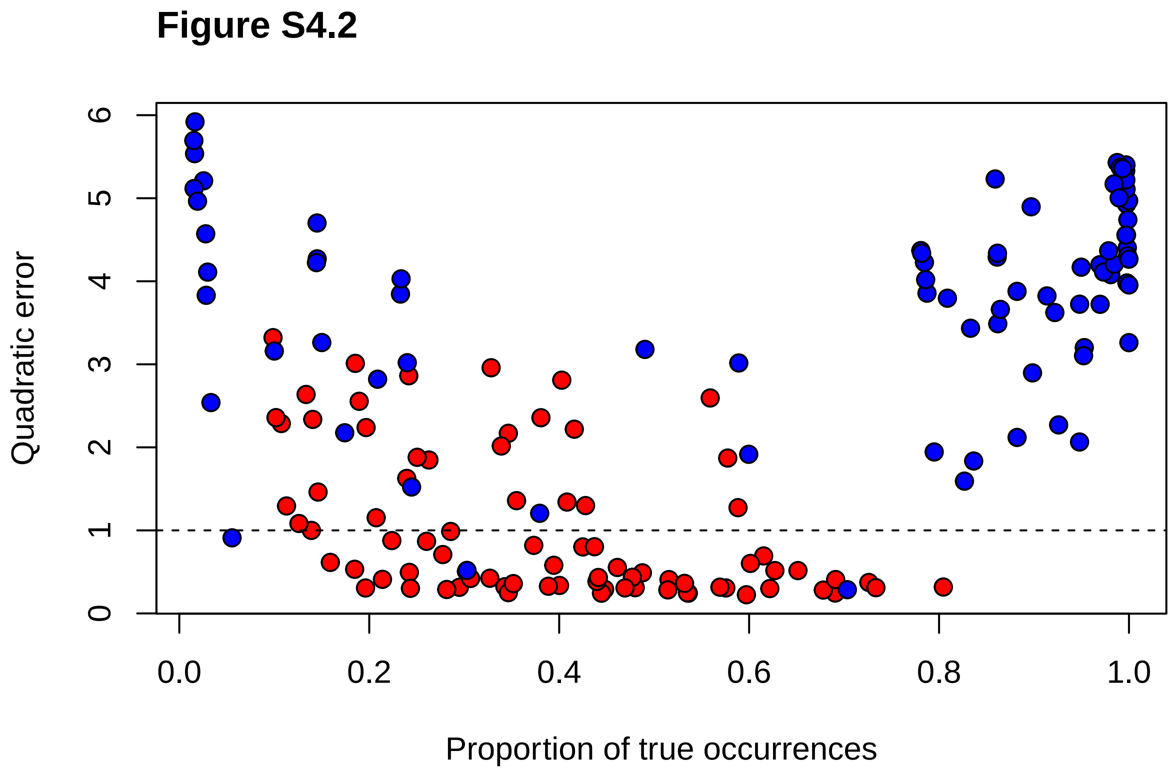

Figure S4.2: Quadratic error as a function of the proportion of truly occupied sites in the dataset for the two arrays of parameters. *Red (resp. blue) points correspond to parameters obtained with $log(2)$ (resp. $log(10)$) shifts from the reference parameter set.*

Proportion of true occupancies were more intermediary for parameter sets obtained by $log(2)$ shifts from the reference log-parameter set, thus rendering estimation possible. Then the quadratic error was distributed between 0.2248129 and 3.3208083, with more than half of the parameter sets yielding a quadratic error below 1 (Figure S4.3). We reckoned that 1 is a meaningful threshold for QE since $QE < 1$ means that the four parameter estimates necessarily have the same order of magnitude than the corresponding true value (i.e. difference below 1 on a log scale).

Figure S4.3

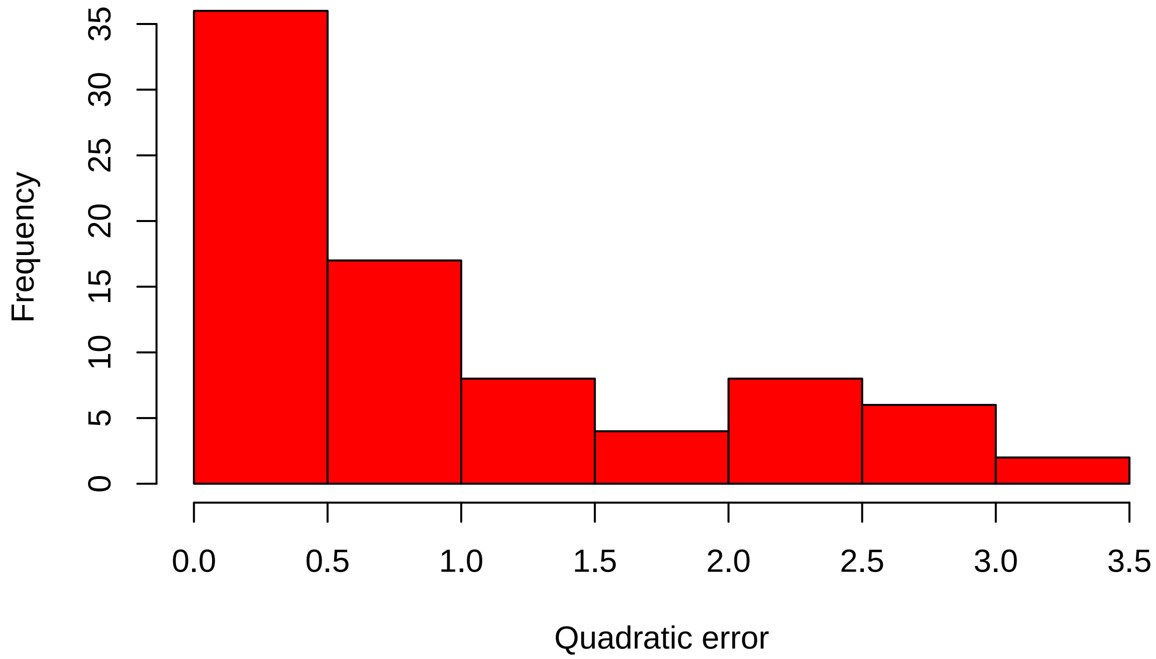

Figure S4.3: Distribution of quadratic error for the parameter sets obtained with $log(2)$ shifts from the reference parameter set.

A correlation analysis (Figure S4.4) showed that among parameter sets obtained by log(2) shifts, those that yielded the highest QEs were those with low C and high α, that is weak colonization with strong distance limitation. This is consistent with the fact that there is a negative relationship between QE and proportion of true occupancies across parameter sets obtained with log(2) shifts (for the same reason as the difference in QEs between parameters obtained through log(2) versus log(10) shifts; Figure S4.1).

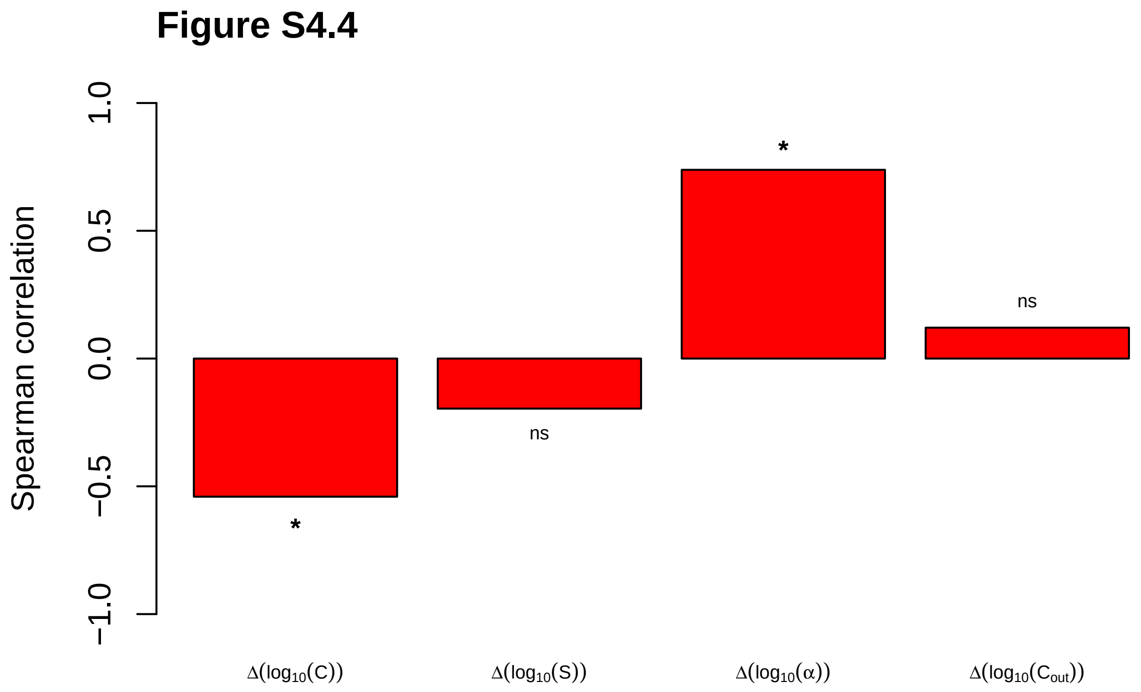

Figure S4.4: Spearman correlation of quadratic error with true parameter values (each parameter considered separately) for parameter sets obtained with $log(2)$ shifts from the reference parameter set. *Stars symbols indicate a significant reject of Spearman rank correlation test with p<0.001, ns means no significat rank correlation (p>0.05).*

Bias and variance/covariance at one parameter set

We now explore in depth the two underlying component of estimation QE, bias and variance, in the particular case where the reference set of parameters (Table 4 of main text) was used to generate the virtual data. In the previous section, we had obtained 200 simulated datasets with the reference parameter set (100 per array), and thus 200 estimates. We observed that estimates were slightly but significantly negatively biased, except for $log_{10}(c)$ estimate which was not significantly biased in our study (Figure S4.5).

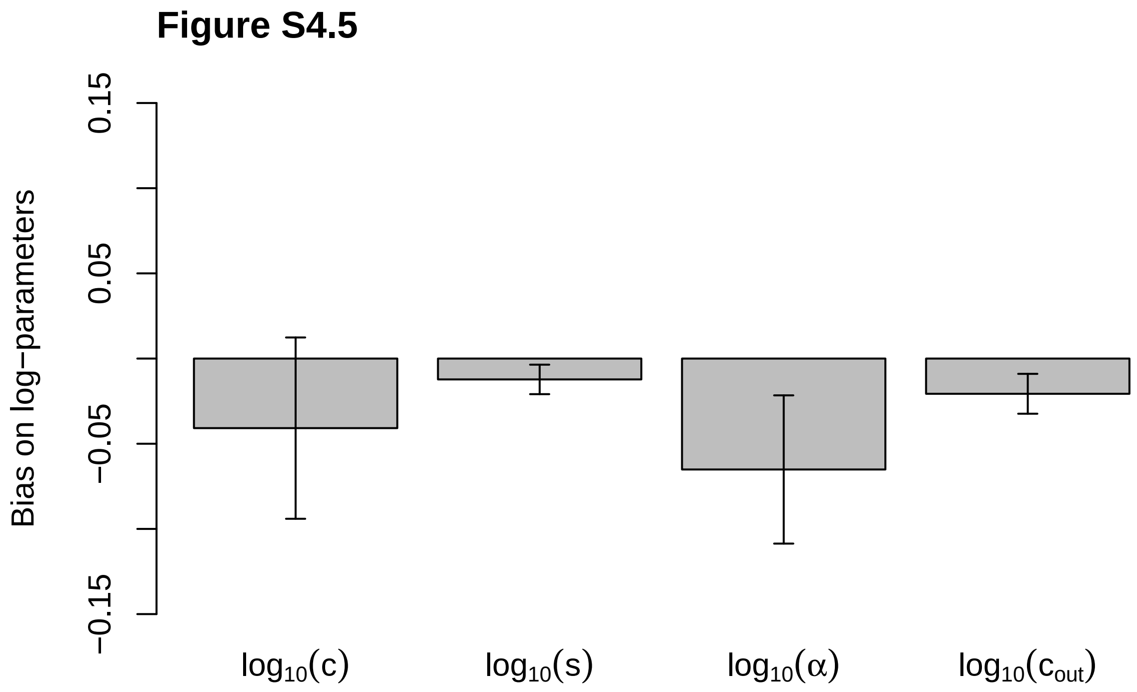

Figure S4.5: Bias of estimates for simulated data with the reference parameter set. * Error bars indicate a 95% confidence interval of the bias.*

Standard deviation of estimates explained most of the quadratic error of estimation (Figure S4.6).

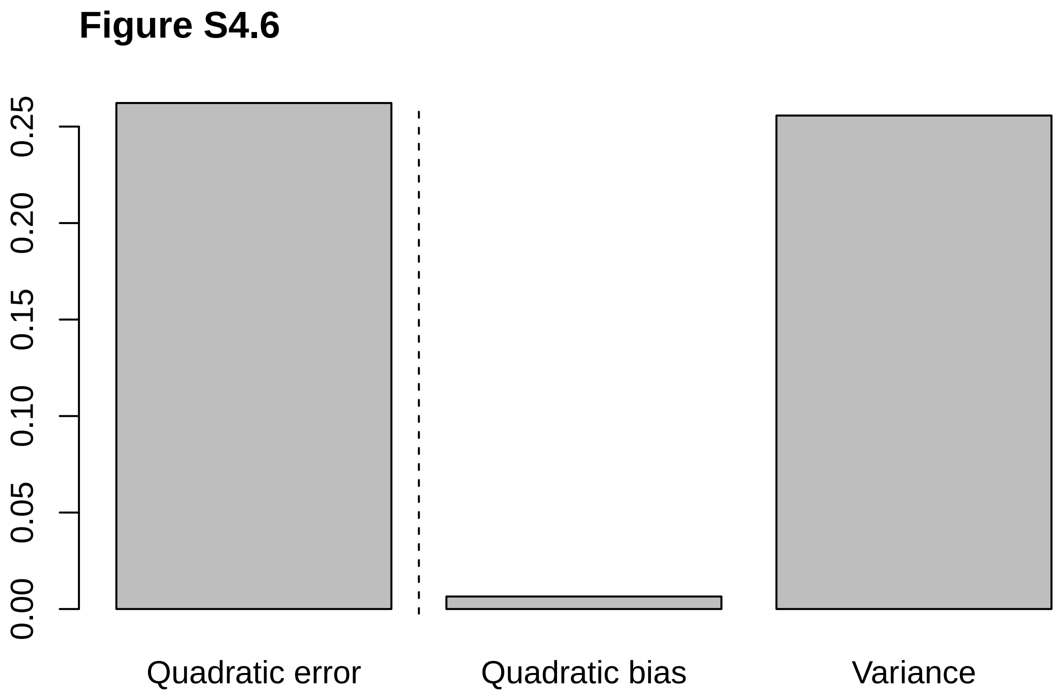

Figure S4.6: Decomposition of quadratic error into quadratic bias and estimation variance, obtained from simulation with the reference parameter set. *The sum of the second and third bars equals the first bar.*

The estimates of $log_{10}(c)$ and $log_{10}(\alpha)$ were more variable that those of $log_{10}(s)$ and $log_{10}(c_{out})$ (Figure S4.7). This stems from the fact the benefit of a higher $log_{10}(c)$ on colonization ability is partially dampened by the negative effect of a higher $log_{10}(\alpha)$. As a result, many couples of $(log_{10}(c), log_{10}(\alpha))$ along some positive relationship are hard to distinguish from occupancy data, hence the large standard deviations. One may call this phenomenon a "weak identifiability constraint".

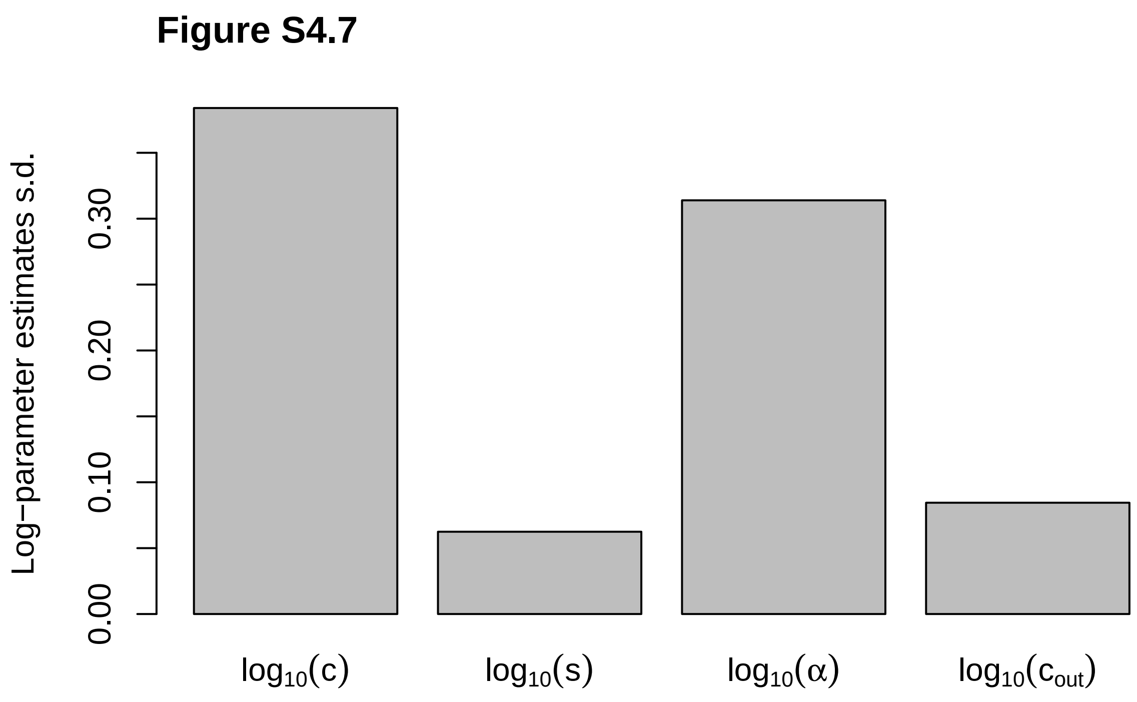

Figure S4.7: Standard deviation of estimates for simulated data with the reference parameter set.

As another sign of this weak identifiability constraint, we observed a strong positive correlation between $log_{10}(c)$ and $log_{10}(\alpha)$ estimates:

```
##                   log[10](c)  log[10](s) log[10](alpha) log[10](c[out])
## log[10](c)         1.0000000 -0.19204582     0.94965459       0.4235985
## log[10](s)        -0.1920458  1.00000000    -0.09059164      -0.3487449
## log[10](alpha)     0.9496546 -0.09059164     1.00000000       0.5527973
## log[10](c[out])    0.4235985 -0.34874492     0.55279733       1.0000000
```

Summary of results regarding estimation quality

- the quadratic error of our procedure is below 1 as long as parameters do not lead to a "saturated" occupancy state (all the sites occupied or all the sites devoid of individuals);
- we detected negative bias on three our of four parameters, but it is weak and does not contribute much to the quadratic error;
- we detected a strong correlation between two parameter estimates, which we reported in main text (Table 4).
