# Supplementary material for: Abundance-based detectability in a spatially-explicit metapopulation: a case study on a vulnerable beetle species in hollow trees
Source: Oecologia. 2018 Jul 31;188(3):671–82. doi: 10.1007/s00442-018-4220-5 (PMC6208700; doi:10.1007/s00442-018-4220-5)
Supplement: Supplementary file 5 — Supplementary material 5 (PDF 102 kb) [file 442_2018_4220_MOESM5_ESM.pdf]

Online Resource 5: Sensitivity of selected model and parameter estimates to dispersal kernel and abundance-based carrying capacity

F. Laroche, H. Paltto, T. Ranius

Contents

Changing dispersal kernel to a fat-tail function **1**

Neglecting trees carrying capacity **2**

Changing dispersal kernel to a fat-tail function

The strong unstructured colonization in our analysis may be due to long-distance dispersal within our study area, which would be poorly captured by our thin tail dispersal kernel (exponential). However, such an effect should bend the metapopulation model selection towards a Levins model (strong c and low α) rather a strong unstructured colonization c_{out}, which is not what is observed here. However, we provide here a double check by performing the model selection and estimation again using a fat-tail dispersal kernel. This was done by replacing equation (6) of main text by:

$$\rho_{i,j,t} = \left[1 - exp\left(-\frac{cK_j}{1 + \left(\alpha d_{ij}\right)^2}\right)\right] \tag{1}$$

All the parameters keep the same interpretation. Running the estimation procedure for the full model and the four sub-models yields following pseudo-AIC:

```
## [1] "Prop. rain : 924.660744983595"

## [1] "Closed LM : 928.333602539492"

## [1] "Closed SRLM : 929.807542901904"

## [1] "Open LM : 926.660746822603"

## [1] "Open SRLM : 929.277789986558"
```

We observe that the best model with the fat-tail dispersal kernel is the "propagule rain" model. In other words changing the kernel made us lose track of local colonization events. In addition, the AIC of the best model is now 925, which is by far higher that the AIC of the best model with the exponential kernel (full model; AIC=914). At last, the "propagule model" selected here yields an unstructured colonization parameter c_{out} equal to 0.2112339, which is higher than the coefficient of the selected model in main text (c_{out}=0.1467118).

Consequently, switching to a fat-tail dispersal kernel did not decrease but increase the importance of unstructured colonization, thus further discarding the idea that our unstructured colonization signal could stem for inadequate choice of local dispersal kernel.

Neglecting trees carrying capacity

Because our observation model has a limited R^2, it is reasonable to check whether our results are not entirely relying on carrying capacity estimates, which would make their interpretation delicate. Consequently, we ran again estimation while neglecting heterogeneity among trees in terms of carrying capacity. To do so, we replaced the carrying capacities obtained with equation (3) of main text by a unique carrying capacity K computed as:

$$K = -log\left(\frac{1}{N}\sum_{i=1}^{N}\frac{e^{\mu+\theta_i}}{1+e^{\mu+\theta_i}}\right) \tag{2}$$

where notations are identical to equation (3) of main text. We also ignored detectability using the method described in main text, because it also depends on our observation model. Running again the estimation procedure for the full model and the four sub-models yields following pseudo-AIC:

```
## [1] "Prop. rain : 944.062922619636"
## [1] "Closed LM : 948.756148597105"
## [1] "Closed SRLM : 949.892143025172"
## [1] "Open LM : 946.062991749713"
## [1] "Open SRLM : 934.055584618576"
```

The best model is still the full model ("Open SRLM"), with an AIC much lower than any sub-models. In addition, the AIC of the full model with constant tree carrying capacity and no detectability is 5 points higher that the model with heterogeneous trees carrying capacity and no limited detectability that we presented in main text (AIC = 929; see Table 3).

Consequently, eliminating limited detectability and heterogeneous tree carrying capacity did not change the outcome of model selection. In addition, it lead to a worse fit than when including tree carrying capacity (-5 pts in AIC), which in turn lead to a worse fit than when also including limited detectability (- 15 pts in AIC).

When ignoring heterogeneous carrying capacity and limited detectability, we obtained the following estimates:

```
##      log10(c)      log10(s)  log10(alpha) log10(c[out])
##   -0.99330234   -0.07447835    1.41812487   -0.83353489
```

with corresponding standard deviations:

```
##      log10(c)      log10(s)  log10(alpha) log10(c[out])
##    0.38454292    0.06507635    0.31443008    0.13209448
```

These estimates are not significantly different from those obtained when including heterogeneous trees carrying capacity. We also assessed the correlation among (log-) estimates using parametrical bootstrap. We obtained the following correlation matrix:

```
##                  log10(c)    log10(s) log10(alpha) log10(c[out])
## log10(c)       1.00000000  0.02564349   0.93380886     0.3098906
## log10(s)       0.02564349  1.00000000   0.05256937    -0.2786959
## log10(alpha)   0.93380886  0.05256937   1.00000000     0.5561208
## log10(c[out])  0.30989064 -0.27869592   0.55612084     1.0000000
```

The correlation matrix obtained is very similar to that of Table 4 in main text. In particular, we retrieved the high degree of positive correlation between α and c estimates, which suggests that data is insufficient to clearly disentangle their antagonistic effects.
